# Yaobishu Regulates Inflammatory, Metabolic, Autophagic, and Apoptosis Pathways to Attenuate Lumbar Disc Herniation

**DOI:** 10.1155/2022/3861380

**Published:** 2022-05-14

**Authors:** Xiaosheng Li, Shuoqi Li, Zhengwu Zang, Yinhao He

**Affiliations:** ^1^Department of Arthrology, Hunan Provincial People's Hospital, Changsha 410001, China; ^2^Department of Orthopaedics, The Second Xiangya Hospital of Central South University, Changsha 410001, China

## Abstract

**Objective:**

Here, we aimed to explore the main mechanism of Yaobishu (YBS) in lumbar disc herniation (LDH).

**Methods and Results:**

Eighteen compounds that might act on LDH were obtained through a combination of network pharmacology prediction and identification by high-performance liquid chromatography-mass spectrometry. The key compounds were palmitic acid and trans-4-hydroxy-3-methoxycinnamate (cinnamate). KEGG analysis demonstrated that palmitic acid target genes mainly regulate the PPAR signaling pathway, Ras signaling pathway, and fatty acid metabolism. Cinnamate target genes were primarily involved in chemical carcinogenesis-receptor activation, lipid and atherosclerosis, the HIF-1 signaling pathway, and nitrogen metabolism. The rat LDH model was constructed using autologous nucleus pulposus tissue implantation. Differential expression gene (DEGs) related to metabolism (CDKN1A and UHRF1), inflammation (S100A9 and SOCS3), autophagy (DCN and LEPR), and apoptosis (CTSW and BCL2A1) in dorsal root ganglion (DRG) tissues of the control and LDH groups was evaluated by RNA-Seq. TNF-*α* stimulated DRG neuronal cells were used to establish an *in vitro* LDH model. YBS, palmitic acid, and cinnamate reduced the expression of substance P, CGRP, S100A9, CTSW, and cleaved caspase-3, while enhancing the expression of CDKN1A, UHRF1, PCNA, Ki67, SOCS3, DCN, LEPR, and BCL2A1, as well as telomerase activity. Pearson's correlation analysis confirmed that DCN was positively correlated with BCL2A1, indicating that autophagy might be negatively correlated with apoptosis in LDH. YBS, palmitic acid, and cinnamate reduced the Siegal neurological score and serum IL-1*β* and IL-18 levels, while increasing changes in the hind paw mechanical withdrawal threshold. The RNA-Seq results further showed that YBS downregulated S100A9 and CTSW expression, while upregulating SOCS3, CDKN1A, UHRF1, DCN, LEPR, and BCL2A1 expression.

**Conclusion:**

YBS and its compounds, palmitic acid, and cinnamate, attenuated LDH by regulating the inflammatory, metabolic, autophagic, and apoptotic pathways. Our results might improve the theoretical and experimental basis for clinical applications of LDH disease treatment.

## 1. Introduction

Lumbar disc herniation (LDH) is a common clinical spinal disease that severely reduces patients' quality of life [1]. Discectomy and conservative treatment are currently commonly used effective treatments for LDH in clinical practice [2, 3]. Discectomy has several medical and surgical complications, including infection, durotomy, nerve injury, symptomatic reprotrusion, and secondary surgical injury [4]. Traditional Chinese medicine is a conservative treatment method for LDH that has been proven to effectively relieve the symptoms of LDH [5, 6]. At present, the pharmacological mechanism of traditional Chinese medicine for the treatment of LDH remains poorly understood. Therefore, an in-depth study of the molecular mechanisms of traditional Chinese medicine is necessary to treat LDH.

LDH is mainly caused by ring fiber breakage and discrete leakage of the nucleus pulposus compressing nerves, leading to back pain and radiating pain [7]. Studies have shown that LDH is related to inflammation and metabolism in nucleus pulposus cells [8]. Inflammation in the context of lower back pain affects the progression of LDH [9]. As previously described, the viability of dorsal root ganglion (DRG) neurons in LDH decreased, and apoptosis and inflammation were enhanced [10]. Activating the RAGE/STAT3 pathway in the DRG contributes to persistent pain hypersensitivity caused by LDH [11]. In addition, pharmacological activation of AMPK in DRG neurons mitigated LDH-induced pain-allergic behavior by regulating mTOR/p70S6K signaling [12]. Therefore, LDH progression might be closely related to DRG tissue metabolism, inflammation, autophagy, and apoptosis.

At present, traditional Chinese medicine is commonly used clinically to treat LDH, such as Yaobitong capsule (different from Yaobishu decoction) [13] and Shentong Zhuyu decoction [14]. As previously described, dexamethasone compounds alleviate nerve root pain in LDH rats by inhibiting the inflammatory response of the DRG tissues [15]. Curcumin compounds inhibited LDH radiculopathy by reducing DRG inflammation and apoptosis [16]. YQHR inhibited the release of inflammatory factors by activating autophagy, thereby alleviating intervertebral disc degeneration [17]. Previous studies have shown that traditional Chinese medicine can improve LDH by regulating cell metabolism, inflammation, autophagy, and apoptosis.

Yaobishu (YBS) is a traditional Chinese medicine decoction with a definite clinical effect in treating severe LDH. However, its specific mechanism of action remains poorly understood. It is unclear whether YBS could alleviate the symptoms of LDH by regulating metabolism, inflammation, autophagy, and apoptosis in DRG neurons. In this study, we attempted to identify the active ingredients of YBS using high-performance liquid chromatography-mass spectrometry (HPLC-MS). Next, the potential active ingredients of YBS in LDH disease were predicted using network pharmacology. The key compound of YBS acting on LDH was obtained by taking the intersection of the predicted and identified compounds. We combined *in vivo* and *in vitro* LDH models to explore the potential molecular mechanisms of YBS and its key compounds in LDHs. Our findings suggest a new treatment strategy for LDH.

## 2. Materials and Methods

### 2.1. HPLC-MS Analysis

The main components of YBS were *Homalomena occulta* 25 g, *Astragalus membranaceus* 40 g, *Angelica dahurica* 15 g, *Callerya nitida* 25 g, and *Angelica sinensis* 25 g. The preparation room was prepared according to the prescribed protocol, and the extract was concentrated. The traditional Chinese medicine was provided by the Traditional Chinese medicine Pharmacy of Hunan Provincial People's Hospital. A concentrated sample (200 *μ*L) was added to 800 *μ*L of deionized water and centrifuged at 12000 r/min for 10 min. The supernatant was filtered through a 0.45 *μ*m membrane. About 20 *μ*L samples were subjected to HPLC analysis (LC-30; Shimadzu, Tokyo, Japan). HPLC was used to scan the maximum absorption wavelength of the YBS sample using a PDA detector. The specific HPLC parameters were as follows: the chromatographic column was a SHIMADZU InerSustain C18 (100 × 2.1 mm, 2 *μ*m) (Agilent Technologies, Santa Clara, USA). The mobile phase consisted of organic phase A (acetonitrile) and aqueous phase B (0.1% formic acid). The chromatographic conditions are listed in Table 1. The column temperature was 35°C with a flow rate of 0.300 mL/min.

Hybrid quadrupole-TOF LC/MS was used for the qualitative analysis. Electrospray ionization (ESI) positive and negative ion modes were used for detection. The ESI source conditions were as follows: ion source gas 1, 50; ion source gas 2, 50; curtain gas, 25; source temperatures, 500°C (positive ion) and 450°C (negative ion); ion spray voltage floating (ISVF), 5500 V (positive ion) and 4400 V (negative ion); and TOF MS scan range, 100-1200 Da; product ion scan range, 50-1000 Da. The TOF MS scan accumulation time was 0.2 s with a product ion scan accumulation time of 0.01 s. The secondary mass spectrum was acquired using information-dependent acquisition (IDA), and a high-sensitivity mode was adopted. The declustering potential (DP) was ±60 V, and the collision energy was 35 ± 15 eV.

### 2.2. Network Pharmacology Analysis

First, the ingredients of the YBS Chinese medicine were prepared. The ETCM database predicts the compounds contained in each Chinese medicine component and their corresponding chemical formulas. The ETCM database denotes the compound contained in each Chinese medicine component and the identified compound to take the intersection. The network diagram was drawn using the graph package (version 1.2.6) of R software (version 4.0.2). Two key compounds were obtained: palmitic acid and trans-4-hydroxy-3-methoxycinnamate (cinnamate). The ETCM database predicted the target genes of palmitic acid and constructed a network diagram. The SMILES ID of cinnamate was obtained from the PubChem database (https://pubchem.ncbi.nlm.nih.gov/) [18] and imported into the SwissTargets database (http://www.swisstargetprediction.ch/) [19] to obtain the target of cinnamate. Second, the cinnamate target was searched using the GeneCards database (https://www.genecards.org/) [20] and the HERB database (http://herb.ac.cn/) [21]. The obtained targets were calibrated using the UniProt database (https://www.uniprot.org/) [22]. In total, 158 targets were identified after deduplication. A network diagram was constructed using Cytoscape (version 3.8.0). The target was subjected to Gene Ontology (GO) and Kyoto Encyclopedia of Genes and Genomes (KEGG) enrichment analyses. GO included biological process (BP), molecular function (MF), and cell component (CC). The String database was quoted, and items with a corrected *P* value < 0.05 were selected. R software (version 4.0.2) was applied. Histograms and bubble charts were drawn using the cluster profile, enrichment plot, and ggplot2 packages.

### 2.3. RNA-Seq

Total RNA was isolated from rat DRG tissues using a Qiagen RNeasy Mini Kit (Qiagen, GmbH, Hilden, Germany). The enriched mRNA was reverse-transcribed to form double-stranded cDNA. After purification, the library was constructed and sequenced using the Illumina TruSeq RNA Library Prep Kit, Illumina HiSeq2000, and Illumina HiSeq4000. Specific RNA-Seq libraries were then generated. First, we used fastp (https://github.com/OpenGene/fastp) to control for the quality of raw reads offline. Low-quality data were filtered to obtain clean reads. The clean data were compared with the reference genome sequence using HISAT2 to obtain an aligned reference genome. Stringtie software was used to reconstruct the transcripts. Accurate transcript results were obtained by assembly. The expression of each gene or transcript was determined. DESeq2 or edgeR was used for differential expression analysis. Finally, relevant pathways were identified through enrichment analysis (KEGG and GO).

### 2.4. Cell Culture and Intervention

Normal DRG neuronal cells (CP-R126; Procell, China) were cultured in fresh growth medium composed of F-12 medium, 100 U/mL penicillin, and 100 *μ*g/mL streptomycin.

1. To determine the effects of YBS, palmitic acid, and cinnamate on normal cell proliferation, we divided the cells into ten groups. Normal DRG neuron cells in the YBS low-dose (YBS-low) group, YBS-medium-dose (YBS-medium) group, and YBS-high-dose (YBS-high) groups were treated with 1.5 *μ*g/mL, 3 *μ*g/mL, and 6 *μ*g/mL YBS for 2 h at 37°C, respectively. Normal DRG neuron cells in the palmitic acid low dose (palmitic acid-L) group, palmitic acid medium dose (palmitic acid-M) group, and palmitic acid high dose (palmitic acid-H) group were treated with 0.1 *μ*M, 1 *μ*M, and 10 *μ*M palmitic acid (MedChemExpress, USA) for 2 h at 37°C, respectively. Normal DRG neurons in the cinnamate low-dose (cinnamate-L) group, cinnamate-medium-dose (cinnamate-M), and cinnamate-high-dose (cinnamate-H) were treated with 0.1 *μ*M, 1 *μ*M, and 10 *μ*M cinnamate (Shanghai Yuanye Bio-Technology Co., Ltd., China) for 2 h at 37°C, respectively. The cells in the control group were treated with the same amount of vehicle
2. To explore the effects of YBS, palmitic acid, and cinnamate on the proliferation of DRG neurons induced by TNF-*α*, we divided the cells into 11 groups: control, TNF-*α*, YBS-low, YBS-medium, YBS-high, palmitic acid-L, palmitic acid-M, palmitic acid-H, cinnamate-L, cinnamate-M, and cinnamate-H groups. As described in previous studies [1, 2], TNF-*α*-induced DRG neuron cells were used in this study to simulate the in vitro LDH model. Except for the control group, the cells in the other groups were treated with 10 ng/mL TNF-*α* (Cell Signaling Technology, Danvers, MA, USA) and incubated for 48 h at 37°C. Except for the control and TNF-*α* groups, the cells in the other groups were treated according to the concentration described in (1). The cells in the control group were treated with the same amount of the vehicle

### 2.5. Cell Counting Kit-8 (CCK-8)

Cells were seeded in a 96-well plate at a density of 5 × 10^3^ cells/well. The experimental procedure strictly followed the CCK-8 assay protocol (AWC0114a; Abcam, China). After the cells had adhered to the wall, 100 *μ*L of CCK-8 working solution was added to each well. The cells were incubated for 4 h at 37°C with 5% CO_2_. A Bio-Tek microplate reader (MB-530, HEALES, China) was used to analyze absorbance at 450 nm.

### 2.6. Immunofluorescence

Rat DRG tissues were fixed with 4% paraformaldehyde for 24 h. The tissue was dehydrated using an alcohol gradient. Xylene was used to ensure tissue transparency. Then, the tissues were then embedded in paraffin and cut into 2-3 *μ*m sections. The slices were baked at 62°C for 8 h. The sections were then dewaxed and rehydrated. Next, 0.01 M citrate buffer (pH 6.0) was used to perform antigen retrieval on the sample in a microwave oven (MM721NG1-PS; MIDEA, China). The samples were sealed with 5% BSA at 37°C for 1 h. The samples were incubated with primary antibodies at 4°C overnight, including DCN (sc-73896, Mouse, 1 : 50, USA) and BCL2A1 (PA5-20268, Rabbit, 1 : 50, USA). Phosphate buffer solution (PBS) (pH 7.4) was used to replace the primary antibody as a negative control. The following day, the samples were incubated with fluorescently coupled secondary antibodies, including goat anti-mouse AF594 (AWS0004a; Abiowell, China) and goat anti-rabbit AF488 (AWS0005a; Abiowell, China), for 1 h at room temperature. DAPI staining solution (AWC0291a; Abiowell, China) was incubated with the tissue at 37°C for 10 min. After mounting the slides in buffered glycerol, tissue staining was observed under a fluorescence microscope (BA410T; Motic, Singapore).

The cell slides were fixed with 4% paraformaldehyde for 30 min. The cell slides were washed three times with PBS for 5 min each time. The cell slides were incubated with 0.3% triton-100 at 3°C for 30 min. Then, 5% BSA was used to block the cells for 60 min at 37°C. The diluted primary antibodies were added to the cells overnight at 4°C, including NeuN (ab177487; rabbit, 1 : 100, UK), DCN (sc-73896; mouse, 1 : 50, USA), and BCL2A1 (PA5-20268; rabbit, 1 : 50, USA). PBS buffer was used to replace the primary antibody as a negative control. The following day, the cells were dropped with 50-100 *μ*L of fluorescent secondary antibody and incubated at 37°C for 90 min, including goat anti-rabbit AF594 (AWS0006a; Abiowell, China), goat anti-mouse AF594 (AWS0004a; Abiowell, China), and goat anti-rabbit AF488 (AWS0005a; Abiowell, China). DAPI staining solution (AWC0291a; Abiowell, China) was incubated with the cells at 37°C for 10 min. After mounting the slides in buffered glycerol, cell staining was observed under a fluorescence microscope (BA410T; Motic, Singapore).

### 2.7. Flow Cytometry

The cells were centrifuged at 1500 rpm for 5 min. PBS (300 *μ*L) was then added to resuspend the cells. The kit (ab65613, UK) was applied to measure cleaved caspase-3. The experimental procedure strictly followed the manufacturer's instructions. Briefly, 1 *μ*L of FITC-DEVD-FMK and 3 *μ*L of propidium iodide were added to the cells. After mixing, the cells were incubated at room temperature for 30 min. The cells were centrifuged at 3000 rpm for 5 min. The washing buffer (300 *μ*L) was added to the cell precipitate to resuspend the cells. The cells were then analyzed using a flow cytometer (A00-1-1102; Beckman, USA).

### 2.8. Quantitative Real-Time Polymerase Chain Reaction (qRT-PCR)

Total RNA was isolated from the DRG tissues in each group using TRIzol® reagent (15596026; Thermo Fisher, USA). cDNA was synthesized using an mRNA reverse transcription kit (CW2569; CWBIO, China). UltraSYBR Mixture (CW2601; CWBIO, China) was used for PCR. The fluorescent quantitative PCR system used was a Thermo Fisher (PIKOREAL96). *β*-Actin was used as the internal reference. The relative expression levels were calculated using the 2^-*ΔΔ*Ct^ method. The primer sequences (Sangon Biotech, Shanghai, China) are listed in Table 2.

### 2.9. Western Blotting

An appropriate amount of radioimmunoprecipitation assay (RIPA) lysate (P0013B; Beyotime, China) was added to the tissues and cells. After centrifugation, cell supernatants were collected. A BCA protein quantification kit was used to determine the protein concentration. Each group had the same mass of protein and was transferred to a Blot Bis-Tris gel. After electrophoresis, proteins were transferred to a membrane. The membrane was immersed in a 5% blocking solution and sealed at room temperature for 1 h. The appropriate amount of primary antibody was incubated with the membrane for 90 min at room temperature, including CDKN1A (10355-1-AP; rabbit, 1 : 1000, USA), UHRF1 (ab251181, rabbit, 1 : 1000, UK), PCNA (10205-2-AP; rabbit, 1 : 5000, USA), Ki67 (ab16667; rabbit, 1 : 1000, UK), SOCS3 (66797-1-Ig; mouse, 1 : 3000, USA), S1000A9 (26992-1-AP; rabbit, 1: 1000, USA), DCN (14667-1-AP; rabbit, 1 : 2000, USA), LEPR (20966-1-AP; rabbit, 1 : 2000, USA), CTSW (ab191083; rabbit, 1: 20000, UK), and BCL2A1 (ab33862; rabbit, 1 : 500, UK). *β*-Actin (66009-1-Ig; mouse, 1 : 5000, USA) was used as an internal reference. HRP-conjugated goat anti-mouse IgG (SA00001-1, 1 : 5000, USA) or HRP-conjugated goat anti-rabbit IgG (SA00001-2, 1 : 6000, USA) was used as the secondary antibody, and the membrane was incubated for 90 min at room temperature. The immunoreactive bands were determined using a chemical imaging agent, and the protein level of each sample was evaluated using Image 6.0.

### 2.10. Animal Model Construction

Eight-week-old SPF Sprague-Dawley rats (*n* = 30) were housed in standard laboratory cages and allowed free access to food and water. Animals were divided into sham, LDH, YBS, palmitic acid, and cinnamate groups (*n* = 6). Except for the sham group, all rats used the LDH model [16, 23–25]. Briefly, the rats were anesthetized via an intraperitoneal injection of sodium pentobarbital (50 mg/kg). The hair on the back of the rat was shaved and routinely disinfected. The intervertebral discs of L5-L6 on the back of the rat were cut longitudinally. The skin and subcutaneous tissues were separated sequentially. The left paraspinal muscles were stripped. The L5-L6 lamina on the left was exposed under a microscope. The lamina, articular process, and part of the pedicle on the left side of L5-L6 were removed. The left dura mater and nerve roots were exposed. The skin and subcutaneous tissue near the root on the ventral side of the rat's tail were cut longitudinally. The coccygeal bone surface was exposed. The separation of the two intervertebral spaces was revealed. The intervertebral disc nucleus pulposus tissue (approximately 5 mg) between the second and third caudal intervertebral discs was harvested under a microscope and gently placed on the exposed nerve roots of L5-L6. The wound was sutured layer by layer. Rats in the sham group were only exposed to the nerve roots of L5 and L6. After the caudal nucleus pulposus was removed, the incision was sutured layer-by-layer. After the rats were fully awake, they were sent to the animal room for normal feeding. Rats were treated with YBS (10 mL/kg/day), palmitic acid (30 mM/kg/day), and cinnamate (30 mM/kg/day) through gastric gavage treatment after LDH surgery. It was administered twice a day for 21 days. At the end of the behavioral test, the rats were intraperitoneally injected with 50 mg/kg sodium pentobarbital. After deep anesthesia, the rats were sacrificed by cervical dislocation. Animals had no spontaneous breathing for 2-3 min and no blink reflex. The DRG tissues and blood samples were collected for follow-up studies. All experimental protocols were approved by the Animal Ethics Committee of Hunan Provincial People's Hospital.

### 2.11. Siegal Neurological Score

The six-level classification method recommended by Siegal was used to assess neurological function: level 0, normal; level 1, basically normal gait and abnormal toes; level 2, weakness of the left hind limb and mild claudication; level 3, weakness of the left hind limb and obvious claudication; level 4, unsteady standing and movable left hind limb; and level 5, paralysis and immobile left hind limb autonomously. Animal behavior was observed on days 0, 3, 7, 14, 21, and 28 after modeling. Statistical analysis was performed using the neurological scores. Level 0 was 2 points. Level 1 was 4 points. Level 2 was 6 points. Level 3 was 8 points. Level 4 was 10 points. Level 5 was 12 points.

### 2.12. Changes in Hind Paw Mechanical Withdrawal Thresholds (PWT)

First, the fiber filament pain meter was calibrated. The needle was used to puncture the bottom of the left hindfoot. The rats withdrawing paw, raising paw, or hissing were all positive reactions, which were distinguished by the voluntary movement of the rats. If the rat demonstrated a positive response, a smaller needle was selected. If no positive reaction was observed, a larger needle was used. The highest strength was 15 g. Each test was repeated five times with an interval of approximately 2 min. Finally, if more than three positive reactions were observed, the lowest intensity was regarded as the PWT of the rat. The test was conducted under double-blind conditions.

### 2.13. Enzyme-Linked Immunosorbent Assay (ELISA)

The blood was centrifuged at 1000 × *g* for 20 min at 2-8°C. The supernatant was then collected. ELISA kits (CSB-E08055r, CSB-E04610r; Wuhan Huamei, China) were used to examine the serum concentrations of IL-1*β* and IL-8. An ELISA kit (ml003023; Enzyme-Linked Biology, China) was used to detect telomerase activity in cells. A Bio-Tek microplate reader (MB-530; HEALES, China) was used to detect the absorbance of cells at a wavelength of 450 nm.

### 2.14. Immunohistochemistry (IHC)

The steps were the same as those used for the immunofluorescence. Briefly, after antigen retrieval, the rat DRG tissues were blocked with 1% periodic acid at room temperature for 20 min. The diluted primary antibodies were added to the tissues overnight at 4°C, including CDKN1A (10355-1-AP, Rabbit, 1 : 100, USA), CGRP (PA5-114929, Rabbit, 1 : 100, USA), Ki67 (ab16667, Rabbit,1 : 100, UK), PCNA (10205-2-AP, Rabbit, 1 : 100, USA), substance P (PA5-106886, Rabbit, 1 : 100, USA), and UHRF1 (ab251181, Rabbit, 1 : 100, UK). HRP-conjugated goat anti-rabbit IgG (PV9001, Zhongshan Jinqiao, China) was incubated with the tissue for 30 min at room temperature. A working solution of DAB chromogen was used for specific staining. Nuclei were stained with hematoxylin for 5 min. Next, tissues were subjected to treatment with graded series of alcohol and xylene treatments and sealed with neutral gum. The tissue was observed under an optical microscope (BA410T; Motic, Singapore).

### 2.15. Transmission Electron Microscope (TEM)

The tissues were fixed with 2% glutaraldehyde at 4°C for two days. The samples were then treated with 1% permeate for 30 min. Ethanol (50%, 70%, 80%, and 100%) was used for dehydration. The samples were immersed in 100% acetone/Epon 812 solution, and subsequently, 60 nm ultrathin sections were cut. The sections were stained with 5% uranyl acetate for 30-60 min and then with lead citrate for 10 min. The samples were observed using TEM (H-7650; Hitachi, Tokyo, Japan).

### 2.16. Statistical Analysis

Data are expressed as the mean ± standard deviation (SD). All experiments were independently repeated three times. One-way analysis of variance (ANOVA) or two-way ANOVA was used for multiple group statistical analyses and comparisons. Statistical analysis was performed using the GraphPad Prism software. Statistical significance was set at *P* < 0.05.

## 3. Results

### 3.1. Identification of YBS Formula and Network Pharmacology Analysis

**Figure 1:**
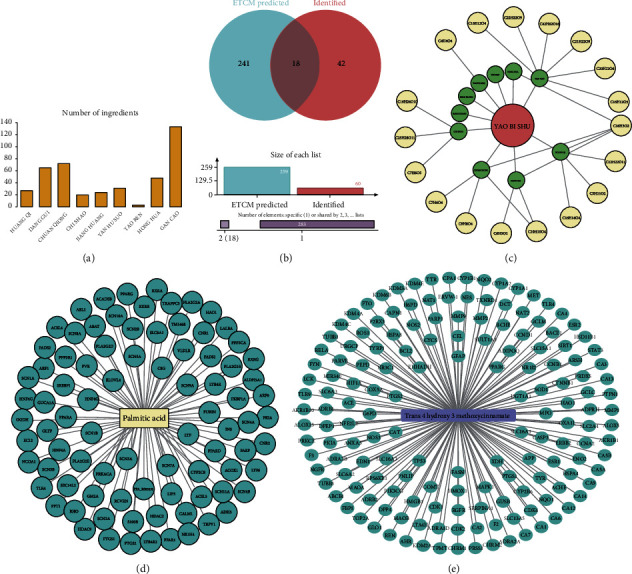
Identification of YBS formula and network pharmacology analysis. (a) The number of Chinese medicine ingredients contained in YBS compound. (b) The intersection of the compound predicted by the ETCM database and the compound identified by HPLC-MS. (c) Network diagram of the intersection compound. The red nodes represent formula. The green nodes represent Chinese herbal medicines. The yellow nodes represent compounds (chemical formula). C16H32O2, palmitic acid. C10H10O4, cinnamate. (d) Palmitic acid target network diagram. (e) Cinnamate target network diagram in LDH. Rectangular nodes represent active compounds and circular nodes represent targets.

Supplementary Figures 1A–1B demonstrated the positive and negative ion current chromatograms of the YBS compound. By comparing with the reference standards, seventy-four main compounds were identified and quantified. The MS qualitative analysis results for the YBS were shown in Supplementary Table 1. The YBS compound contained nine Chinese medicinal ingredients: *Astragalus membranaceus* (HUANG QI), *Angelica sinensis* (DANG GUI), and *Ligusticum chuanxiong* Hort (CHUAN QIONG), *Paeonia anomala* subsp. veitchii (CHI SHAO), *Curcuma longa* (JIANG HUANG), *Corydalis yanhusuo* (YAN HU SUO), *Amygdalus persica* (TAO REN), *Carthamus tinctorius* (HONG HUA), and *Glycyrrhiza uralensis* (GAN CAO) (Figure 1(a)). The ETCM database predicted the compounds contained in each Chinese medicine component and their corresponding chemical formulas. Different compounds might have the same chemical formula, and the naming method of ETCM compounds is different from the naming method of compounds in substance identification results. Therefore, the intersection of chemical formulas was taken to verify whether the chemical formula of interest corresponds to the same compound separately. There were 18 common compounds among those predicted by the ETCM database and identified in our study (Figure 1(b)). A network diagram of 18 common compounds was shown in Figure 1(c). The results suggested that compounds of chemical formula C16H32O2 and C10H10O4 might be the key compounds of YBS. After comparing with the identification results, C16H32O2 was palmitic acid, and C10H10O4 was cinnamate. The ETCM database predicted 85 palmitic acid target genes (Figure 1(d)). Supplementary Figure 2 suggested the GO and KEGG enrichment analysis of the palmitic acid target genes. Palmitic acid target genes were mainly involved in biological processes, such as the regulation of membrane potential, fatty acid metabolic process, action potential, and multicellular organismal signaling. In terms of cell composition, palmitic acid target genes had the highest content of cation channel complexes, ion channel complexes, transmembrane transporter complexes, and transporter complexes. Metal ion transmembrane transporter activity, cation channel activity, gated channel activity, and sodium ion transmembrane transporter activity were the four most important categories of palmitic acid target gene molecular functions. KEGG analysis revealed that palmitic acid target genes were mainly involved in the PPAR signaling pathway, Ras signaling pathway, fatty acid metabolism, and arachidonic acid metabolism. Targets of cinnamate were obtained after calibration and deduplication of the Uniprot database (Figure 1(e)). Supplementary Figure 3 reflected the GO enrichment analysis of cinnamate target genes and the enrichment of the KEGG pathway. Cinnamate target genes mainly regulate the response to oxidative stress, the cellular response to chemical stress, the response to drugs, and the cellular response to oxidative stress. Cinnamate target genes had the highest content in the four cell components of the membrane raft, membrane microdomain, membrane region, and apical part of the cell. The molecular functions of the cinnamate target genes were mainly related to lyase, hydrolase, and carbon-oxygen lyase activities. KEGG analysis reflected that cinnamate target genes regulate chemical carcinogenesis-receptor activation, lipid and atherosclerosis, and chemical carcinogenesis-reactive oxygen species. Through the HPLC-MS experiment combined with the network pharmacology method, we obtained two key compounds of YBS for the treatment of LDH: palmitic acid and cinnamate. Therefore, in this study, we evaluated the effects of YBS, palmitic acid, and cinnamate on DRG neurons and tissues in an LDH model.

### 3.2. RNA-Seq Analysis of Differential Expression Gene (DEGs) in DRG Tissues of LDH Rats and Functional Prediction

**Figure 2:**
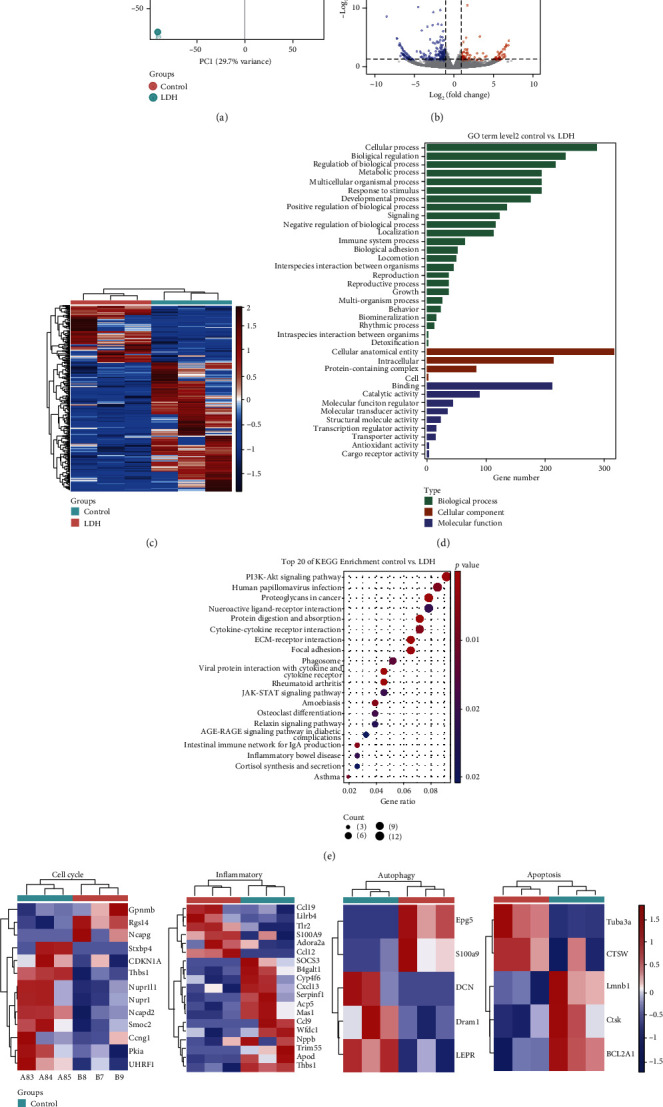
LDH rat DRG tissues RNA-Seq analysis. (a) PCA of DRG tissues in LDH rats. The dot represents the sample. Different colors represent different groups. (b) Differential gene volcano map in DRG tissues of LDH rats. Red dots represent upregulated genes, and green dots represent downregulated genes. (c) Differential gene heat map of DRG tissues in LDH rats. Red indicates high expression, and blue represents low expression. (d) GO functional enrichment analysis of the differential genes. The ordinate is GO term, and the abscissa is the number of differential genes in the GO term. The longer the annotation bar represents, the greater the number of differential genes. (e) Top 20 KEGG enrichment of the differential gene. Red indicates high expression, and blue represents low expression. The ordinate is the pathway, and the abscissa is the enrichment factor (the number of differences in the pathway is divided by all the numbers). The size of the circle indicates the number. The redder the color, the smaller the P or Q value. The redder and the bigger the bubble, the more differential genes are enriched in the pathway. (f) Heat map reflected metabolism, inflammation, autophagy, and apoptosis-related gene expression.

We performed RNA-Seq analysis on the DRG tissues of rats in the control and LDH groups. The principal component analysis (PCA) demonstrated that the two groups of samples were markedly separated (Figure 2(a)). The volcano plot suggested DEGs between the two groups (Figure 2(b)). The heat map revealed DEGs between the two groups (Figure 2(c)). Next, we predicted the functions of DEGs.  Figure 2(d) showed that the DEGs were mainly involved in cellular processes, cellular processes, regulation of biological processes, and metabolic processes. Differential genes were enriched in cellular anatomical entities and in intracellular and protein-containing complexes. The molecular functions of the DEG_S_ were mainly related to binding, catalytic activity, and molecular function regulators.  Figure 2(e) reflected that the DEGs were mainly related to inflammatory pathways, including PI3K-Akt signaling pathway, JAK-STAT signaling pathway, and AGE-RAGE signaling pathway. Based on network pharmacology analysis, KEGG analysis in Supplementary Figure 2 demonstrated that Palmitic Acid target genes participated in PPAR signaling pathway, Ras signaling Pathway, and the thyroid cancer process. KEGG analysis in Supplementary Figure 3 showed that cinnamate target genes regulated AGE-RAGE signaling pathway and progression of various cancer (small cell lung cancer, prostate cancer, bladder cancer, etc.). Cell cycle [26] and apoptosis [27] play an important role in cancer. Therefore, in this study, we investigated whether YBS, palmitic acid, and cinnamate affected LDH progression through inflammation, cell cycle, and apoptosis. Autophagy plays a key role in LDH disease [28]. Yiqi Huoxue prescription might treat LDH by activating autophagy [28]. Therefore, we tried to explore whether YBS, palmitic acid, and cinnamate regulate autophagy gene expression in LDH in this study. Then, we further analyzed the genes related to metabolism, inflammation, autophagy, and apoptosis. We identified 13 metabolism-related DEGs. A total of 18 DEGs were related to inflammation. Five autophagy-related DEGs were also identified. Five DEGs were associated with apoptosis (Figure 2(f)). Among these, CDKN1A and UHRF1 are closely related to metabolic processes [29]. SOCS3 and S100A9 are involved in inflammatory response [30]. DCN and LEPR are associated with autophagy activity [31, 32]. CTSW and BCL2A1 are involved in apoptosis [33]. Therefore, we explored whether YBS and its key compounds affected the expression of the genes mentioned above in the LDH model.

### 3.3. The Effects of YBS and Its Key Compounds on DRG Neuronal Cell Damage and Metabolism *In Vitro*

**Figure 3:**
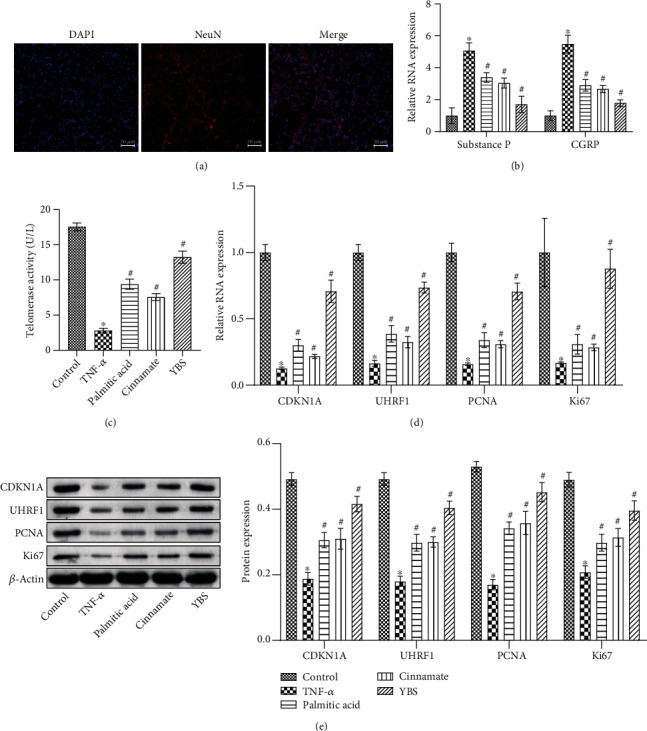
The effect of YBS and its key compounds on the damage and metabolism of DRG neurons induced by TNF-*α*. (a) IHC was applied to measure NeuN expression in cells. (b) qRT-PCR was used to detect the expression of substance P and CGRP in cells. (c) Flow cytometry was used to assess telomerase activity in cells. (d) The expression of CDKN1A, UHRF1, PCNA, and Ki67 in the cells was evaluated by qRT-PCR. (e) Western blotting was used to evaluate CDKN1A, UHRF1, PCNA, and Ki67 expression in the cells. The magnification was 200×. Scale bar, 50 *μ*m. One- or two-way ANOVA was used for multiple group statistical analysis. ^∗^*P* < 0.05 vs. control group, ^#^*P* < 0.05 vs. TNF-*α* group.

We determined that the LDH model led to the differential expression of genes in rat DRG tissues. To determine the effects of YBS and its key compounds on TNF-*α*-induced neuronal cells, we first evaluated the effects of YBS, palmitic acid, and cinnamate on normal neuronal cell proliferation using a CCK-8 assay. The results suggested that different concentrations of YBS, palmitic acid, and cinnamate did not affect cell proliferation, indicating that YBS, palmitic acid, and cinnamate had no toxic side effects on normal neuronal cells (Supplementary Figure 4A). However, YBS, palmitic acid, and cinnamate promoted the proliferation of neuronal cells induced by TNF-*α*, with the medium concentration having the best effect (Supplementary Figure 4B). Next, we measured the number of cleaved caspase-3 positive cells in neuronal cells by flow cytometry. The results showed that YBS, palmitic acid, and cinnamate inhibited neuronal apoptosis induced by TNF-*α*, with the medium concentration having the best effect (Supplementary Figure 4C). Therefore, medium concentrations of YBS, palmitic acid, and cinnamate were selected for subsequent experiments. To identify neuronal cells, we observed the neuron-specific protein NeuN in the cells using immunofluorescence experiments. The results revealed that cell body and neurite morphology were clearly visible, wherein NeuN was widely expressed (Figure 3(a)). Next, we determined the levels of substance P and CGRP mRNA in neuronal cells. The results showed that YBS, palmitic acid, and cinnamate reduced substance P and CGRP expression in TNF-*α*-induced neuronal cells (Figure 3(b)). We used flow cytometry to examine telomerase activity in neuronal cells. The results revealed that the telomerase activity of neuronal cells in the YBS, palmitic acid, and cinnamate groups was higher than that in the TNF-*α* group (Figure 3(c)). Next, we tested the expression of CDKN1A, UHRF1, PCNA, and Ki67 in neuronal cells using qRT-PCR and western blotting. The results suggested that YBS, palmitic acid, and cinnamate increased the expression of CDKN1A, UHRF1, PCNA, and Ki67 in TNF-*α*-induced neuronal cells (Figures 3(d) and 3(e)). Moreover, the efficacy of YBS was better than those of palmitic acid and cinnamate. The effectiveness of the two compounds was similar. In summary, YBS, palmitic acid, and cinnamate inhibited the damage to DRG neurons induced by TNF-*α* and accelerated cell metabolism.

### 3.4. The Effects of YBS and Its Key Compounds on the Inflammatory Response, Autophagy, and Apoptosis of DRG Neurons *In Vitro*

**Figure 4:**
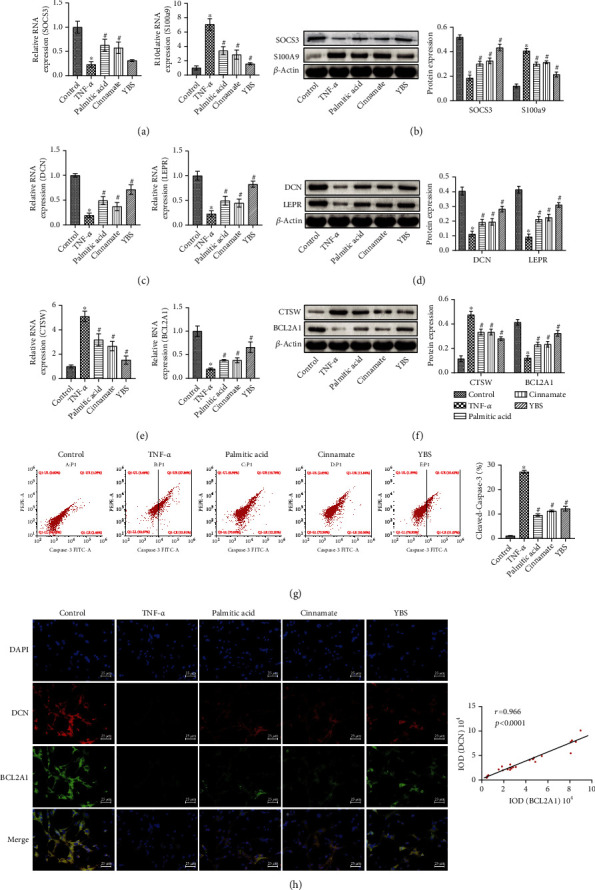
YBS and its key compounds inhibited TNF-*α*-induced inflammation and apoptosis of DRG neuronal cells, and enhanced autophagy. (a) qRT-PCR was used to assess the expression of SOCS3 and S100A9 in cells. (b) Western blotting was used to detect the expression of SOCS3 and S100A9 in cells. (c) Detection of DCN and LEPR expression in cells by qRT-PCR. (d) The expression of DCN and LEPR in cells was detected by western blotting. (e) CTSW and BCL2A1 mRNA expression. (f) CTSW and BCL2A1 protein expression. (g) Flow cytometry to evaluate the expression of cleaved caspase-3 in cells. (h) The colocalization of DCN and BCL2A1 in cells was assessed by immunofluorescence. The magnification was 400×. Scale bar, 25 *μ*m. One- or two-way ANOVA was used for multiple group statistical analysis. ^∗^*P* < 0.05 vs. control group, ^#^*P* < 0.05 vs TNF-*α* group.

Next, we explored the effects of YBS and its key compounds on neuronal inflammation, autophagy, and apoptosis. At the molecular and protein levels, YBS, palmitic acid, and cinnamate improved the expression of SOCS3 in neuronal cells induced by TNF-*α*, while reducing S100A9 expression (Figures 4(a) and 4(b)). Similarly, YBS, palmitic acid, and cinnamate promoted the expression of TNF-*α*-induced neuronal cell autophagy-related factors (DCN and LEPR) (Figures 4(c) and 4(d)). Next, we evaluated the effects of YBS and its key compounds on neuronal cell apoptosis. As shown in Figures 4(e) and 4(f), YBS, palmitic acid, and cinnamate inhibited CTSW expression while promoting the expression of BCL2A1 compared with the TNF-*α* group (Figures 4(e) and 4(f)). The flow cytometry results demonstrated that apoptosis protein cleaved caspase-3 expression was increased in neuronal cells induced by TNF-*α*. YBS, palmitic acid, and cinnamate reduced the expression of cleaved caspase-3 in neuronal cells (Figure 4(g)). Overall, compared with palmitic acid and cinnamate, YBS had more significant anti-inflammatory, antiapoptotic, and autophagy-enhancing effects in TNF-*α*-induced neuronal cells. We had clarified the anti-inflammatory and autophagy-promoting effects of YBS, palmitic acid, and cinnamate in TNF-*α*-induced neuronal cells. Therefore, we used immunofluorescence experiments to evaluate the colocalization of DCN and BCL2A1 in neuronal cells. The results suggested that YBS, palmitic acid, and cinnamate increased DCN and BCL2A1 expression in TNF-*α*-induced neuronal cells. Next, we analyzed the relationship between DCN and BCL2A1 expression using Pearson's correlation. The results showed a positive correlation between DCN and BCL2A1 (*r* = 0.966, *P* < 0.0001), indicating that autophagy might be negatively related to apoptosis in neuronal cells. In summary, YBS, palmitic acid, and cinnamate inhibited inflammation and apoptosis, while enhancing the autophagy of DRG neurons induced by TNF-*α*.

### 3.5. YBS and Its Key Compounds Reduced Siegal Neurological Score and Inflammation and Increased PWT in LDH Rats

**Figure 5:**
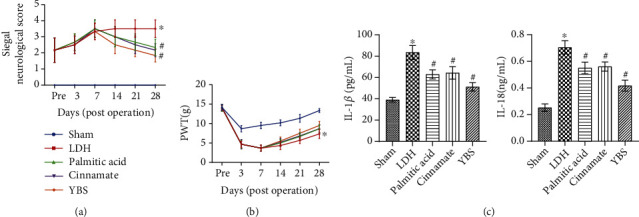
Validation of YBS, palmitic acid, and cinnamate *in vivo*. (a) Siegal neurological score was assessed at different time postoperation (0, 3, 7, 14, 21, and 28 days). (b) PWT was performed 1 day before the operation (pre) and at different time postoperation (3, 7, 14, 21, and 28 days). (c) The serum concentration of IL-1*β* and IL-18 was measured by ELISA. One- or two-way ANOVA was used for multiple group statistical analysis. ^∗^*P* < 0.05 vs. sham group, ^#^*P* < 0.05 vs LDH group.

YBS, palmitic acid, and cinnamate were found to inhibit damage to DRG neuronal cells, reduce inflammation and apoptosis, accelerate metabolic processes, and enhance autophagy. Next, we evaluated the effects of YBS, palmitic acid, and cinnamate on LDH levels *in vivo*. First, we observed the impact of YBS, palmitic acid, and cinnamate on the neurological function of LDH rats through behavioral studies. The results showed that the nerve function of the rats in the sham group was normal. The LDH model increased the neurological function score of the rats, indicating that the neurological function of the rats in the LDH group was damaged. After 7 days after surgery, YBS, palmitic acid, and cinnamate reduced the neurological scores of LDH rats (Figure 5(a)). YBS, palmitic acid, and cinnamate restored nerve function in rats with LDH to a certain extent. We further evaluated rat PWT. The results revealed that the PWT of the preoperative rats was similar, and the PWT of the LDH rats decreased within seven days after the operation. The PWT of rats in the sham group reflected a downward trend within three days after the operation, followed by a gradual upward trend. The PWT of rats in the LDH group was lower than that of rats in the sham group. Seven days after the operation, YBS, palmitic acid, and cinnamate increased the proportion of PWT in LDH rats (Figure 5(b)). Next, we measured the levels of IL-1*β* and IL-18 in the rat serum using ELISA. YBS, palmitic acid, and cinnamate reduced the concentrations of IL-1*β* and IL-18 in the serum of rats with LDH (Figure 5(c)). Our results showed that the therapeutic effect of YBS on LDH in rats was better than that of palmitic acid and cinnamate. In summary, YBS, palmitic acid, and cinnamate restored the neurological score of LDH rats, improved PWT, and inhibited inflammation.

### 3.6. YBS and Its Key Compounds Inhibited DRG Tissue Damage and Metabolic Response in LDH Rats

**Figure 6:**
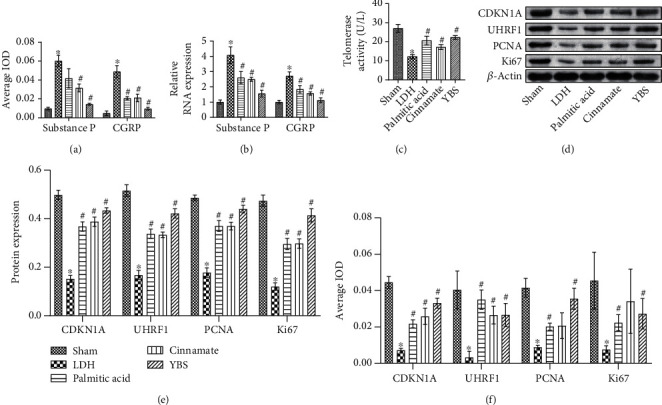
The effects of YBS and its key compounds on DRG tissue damage and metabolic response in LDH rats. (a) Statistical analysis of substance P and CGRP expression in DRG tissues through IHC. (b) qRT-PCR was used to examine the expression of substance P and CGRP in DRG tissue. (c) Telomerase activity in DRG neuron cells was evaluated by flow cytometry. (d) CDKN1A, UHRF1, PCNA, and Ki67 protein expressions in DRG tissue were examined by western blotting. (e) Western blotting protein quantitative statistical analysis in DRG tissues. (f) Statistic analysis of CDKN1A, UHRF1, PCNA, and Ki67 in DRG tissues. One- or two-way ANOVA was used for multiple group statistical analysis. ^∗^*P* < 0.05 vs. Sham group, ^#^*P* < 0.05 vs. LDH group.

Next, we evaluated the effects of YBS, palmitic acid, and cinnamate on DRG tissue damage and metabolic response *in vivo*. The IHC results demonstrated that YBS, palmitic acid, and cinnamate downregulated substance P and CGRP expression in the LDH group (Figure 6(a)). At the molecular level, YBS, palmitic acid, and cinnamate downregulated substance P and CGRP expression in the LDH group (Figure 6(b)). Next, we evaluated telomerase activity in the rat DRG neurons. The results demonstrated that YBS, palmitic acid, and cinnamate promoted telomerase activity in the LDH group (Figure 6(c)). We also assessed CDKN1A, UHRF1, PCNA, and Ki67 expression in DRG tissues using western blotting and IHC. As shown in Figures 6(d) and 6(e), CDKN1A, UHRF1, PCNA, and Ki67 expressions in the YBS, palmitic acid, and cinnamate groups were higher than that in the LDH group. The IHC results were consistent with the western blotting results. YBS, palmitic acid, and cinnamate promoted CDKN1A, UHRF1, PCNA, and Ki67 expression (Figure 6(f) and Supplementary Figure 5). Moreover, YBS was better than palmitic acid and cinnamate in alleviating DRG tissue damage and accelerating metabolic processes in LDH rats.

### 3.7. Anti-Inflammatory, Antiapoptotic, and Enhancing Autophagy Effects of YBS and Its Key Compounds in DRG Tissues of LDH Rats

**Figure 7:**
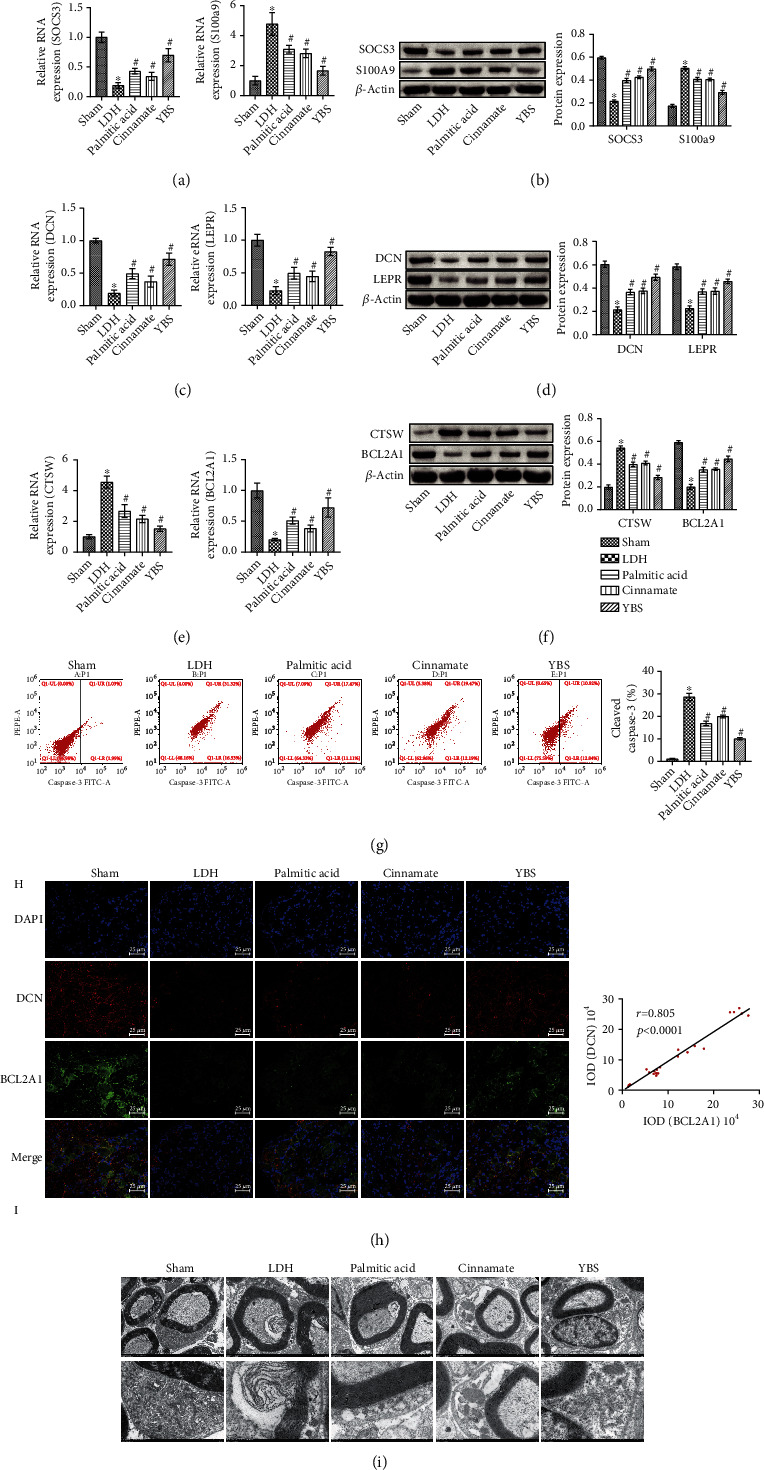
The effect of YBS and its key compounds on the inflammation, apoptosis, and autophagy of DRG tissue in LDH rats. (a) qRT-PCR was applied to assess SOCS3 and S100A9 expression in tissues. (b) The expression of SOCS3 and S100A9 in the tissues was assessed by western blotting. (c) qRT-PCR was applied to detect the expression of DCN and LEPR in tissues. (d) Western blotting was used to measure the expression of DCN and LEPR in the tissue. (e) The expression of CTSW and BCL2A1 in tissues was used by qRT-PCR. (f) Western blotting was used to evaluate the expression of CTSW and BCL2A1 in tissues. (g) Flow cytometry was applied to detect the expression of cleaved caspase-3 in DRG neuron cells. (h) The colocalization of DCN and BCL2A1 in cells was detected by immunofluorescence. (i) TEM was used to observe the changes in cell microstructure in DRG tissue. The magnification was 400×. Scale bar, 25 *μ*m. One- or two-way ANOVA was used for multiple group statistical analysis. ^∗^*P* < 0.05 vs. sham group, ^#^*P* < 0.05 vs. LDH group.

To evaluate the effects of YBS, palmitic acid, and cinnamate on inflammation, apoptosis, and autophagy in the DRG tissues of LDH rats, we performed a series of experiments. First, we used qRT-PCR and western blotting to test SOCS3 and S100A9 expression in the DRG tissues. The results suggested that YBS, palmitic acid, and cinnamate elevated SOCS3 in DRG tissues in LDH rats at the molecular and protein levels while decreasing the expression of S100A9 (Figures 7(a) and 7(b)). Next, we evaluated DCN and LEPR expression in the rat DRG tissues. As shown in Figures 7(c) and 7(d), DCN and LEPR expressions in the DRG tissues of the YBS, palmitic acid, and cinnamate groups were higher than those in the LDH group. We also examined CTSW and BCL2A1 expression in DRG tissues.  Figure 7(e) suggested that YBS, palmitic acid, and cinnamate inhibited CTSW expression in the DRG tissues of LDH rats while promoting BCL2A1 expression. These protein levels were consistent with the molecular-level results. As shown in Figure 7(f), the expression of CTSW in the DRG tissues of the YBS, palmitic acid, and cinnamate groups was decreased compared to that in the LDH group, while the expression of BCL2A1 was increased. In addition, flow cytometry was used to detect the expression of cleaved caspase-3 in rat DRG neurons. The results revealed that YBS, palmitic acid, and cinnamate reduced the number of cleaved caspase-3 positive cells in LDH rats (Figure 7(g)). In general, YBS, palmitic acid, and cinnamate had anti-inflammatory, antiapoptotic, and autophagy-enhancing effects on the DRG tissues of LDH rats. The therapeutic effect of YBS was stronger than those of palmitic acid and cinnamate. We further evaluated the relationship between autophagy and apoptosis in the DRG tissues of LDH rats. The colocalization of DCN and BCL2A1 in DRG tissues was measured by immunofluorescence. The results revealed that YBS, palmitic acid, and cinnamate promoted DCN and BCL2A1 expression in DRG tissues of LDH rats. Pearson's analysis further confirmed a positive correlation between DCN and BCL2A1 (*r* = 0.805, *P* < 0.0001), indicating a negative correlation between DRG tissue autophagy and apoptosis in LDH rats. YBS, palmitic acid, and cinnamate might inhibit the apoptosis of DRG cells in LDH rats by enhancing autophagic activity. Next, we observed changes in the microstructure of cells in DRG tissues using TEM. As shown in Figure 7(i), the intracellular structure in the sham group was stable and orderly. The structure of the myelin sheath in the LDH group was abnormal and similar to dissolution in the local area. The intracellular structure was disordered in the LDH-treated group. Organelles were incomplete in the LDH group. The myelin sheaths of the palmitic acid and cinnamate groups also revealed abnormalities, irregularities, and local tearing. The intracellular structures were arranged out of order and were incomplete. The severity was lighter than the LDH group. The myelin sheath in the YBS group reflected local tearing and an intracellular structure. However, the intracellular structure was partially incomplete. Compared to the palmitic acid and cinnamate groups, the order and completeness of the intracellular structure arrangement in the YBS group were slightly stronger. The TEM results showed that YBS, palmitic acid, and cinnamate alleviated microstructural changes in DRG neurons in LDH rats. In summary, YBS, palmitic acid, and cinnamate inhibited the inflammatory response and cell apoptosis and enhanced autophagy activity in the DRG tissues of LDH rats.

### 3.8. RNA-Seq Analysis of the Effect of YBS on Gene Expression and Function in DRG Tissues of LDH Rats

**Figure 8:**
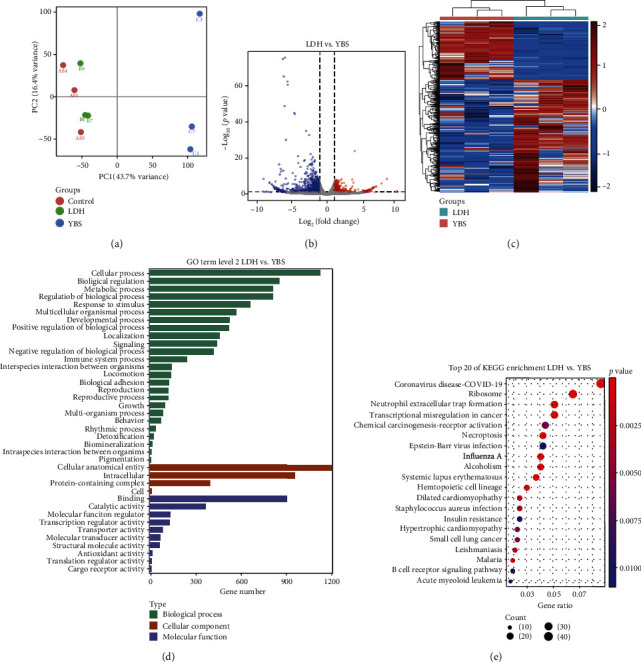
Effect of YBS on gene expression and function in DRG tissues of LDH rats through RNA-Seq analysis. (a) PCA of gene expression in DRG tissue. Dots represent the sample. Different groups are indicated in different colors. (b) Volcano map of DEGs in DRG tissues. Red dots represent genes with increased expression, and green dots represent genes with reduced expression. (c) Heat map of DEGs in DRG tissues. Red indicates high expression, and blue represents low expression. (d) GO terms levels of differentially expressed genes. The ordinate is GO term, and the abscissa is the number of differential genes in the GO term. The longer the annotation bar represents, the greater the number of differential genes. (e) Top 20 KEGG pathway enrichment of DEG. The ordinate is the pathway, and the abscissa is the enrichment factor (the number of differences in the pathway is divided by all the numbers). The size of the circle indicates the number. The redder the color, the smaller the *P* or *Q* value. The redder and the bigger the bubble, the more differential genes are enriched in the pathway.

Next, we performed RNA-Seq analysis of the DRG tissues of rats in the LDH and YBS groups. The PCA indicated that the two sets of samples were separated (Figure 8(a)). DEGs between the two groups were shown in a volcano plot (Figure 8(b)). DEGs between the two groups were presented using a heat map (Figure 8(c)). Next, we predicted the functions of DEGs.  Figure 8(d) demonstrated that DEGs were mainly related to cellular processes, biological regulation, regulation of biological processes, and metabolic processes. In cell composition, the DEGs were enriched in cellular anatomical entities and in intracellular and protein-containing complexes. The molecular functions of DEGs were mainly related to binding, catalytic activity, and molecular function regulators. The GO annotation of DEGs in the LDH and YBS groups was consistent with the GO annotation of DEGs in the control and LDH groups. KEGG analysis demonstrated that the differential genes were mainly related to the coronavirus disease-COVID-19 (Figure 8(e)). We further analyzed the genes related to metabolism, inflammation, autophagy, and apoptosis. We obtained 65 metabolic-related DEGs. There were 47 DEGs related to inflammation, and 20 DEGs were related to autophagy, while 17 DEGs were found to be related to apoptosis. The heat map suggested that YBS inhibited SOCS3 and S100A9 expression. YBS improved CDKN1A, UHRF1, DCN, LEPR, and BCL2A1 expression, while downregulating CTSW expression (Supplementary Figures 6A–6B). Therefore, our findings indicated that YBS might alleviate LDH levels by regulating metabolism, inflammation, autophagy, and apoptosis.

## 4. Discussion

With societal advances and changes in modern lifestyles, the prevalence of LDH is increasing [34]. LDH is the main cause of radiculopathy, and its underlying mechanism remains largely unknown [35]. According to previous reports, traditional Chinese medicine has a definitive effect on the conservative treatment of LDH and can promote the absorption of protrusions [36]. YBS is a traditional Chinese medicine decoction. In this study, we explored the key compounds of YBS in LDH and their mechanisms of action *in vitro* and *in vivo*.

In recent years, the clinical efficacy of YBS in LDH has been confirmed; however, its specific mechanism remains unknown. Ultraperformance liquid chromatography-mass spectrometry (UPLC-MS) and network pharmacology have been widely used to clarify the potential therapeutic mechanisms of Chinese medicine in diseases. For example, network pharmacological analysis found that the active ingredients (ginsenoside Rg1 and ginsenoside Rb1) in the Yaobitong capsule regulated MAPK, Ras, PI3K-Akt, and NF-kappa B signaling pathways through 87 direct target genes to inhibit excessive inflammation in LDH [13]. Based on network pharmacology, Epimedium white peony might play a role in treating LDH through signaling pathways related to inflammation, metabolism, and aging [37]. The eight main compounds of the Huang Gan formula obtained by UPLC-MS analysis might have a renoprotective effect by blocking the Wnt/*β*-catenin signaling pathway [38]. In this study, we first identified the key compounds of YBS for the treatment of LDH based on HPLC-MS combined with network pharmacology analysis, namely, palmitic acid and cinnamate. KEGG functional analysis suggested that palmitic acid target genes mainly regulate the PPAR signaling pathway, Ras signaling pathway, fatty acid metabolism, and arachidonic acid metabolism, among other pathways. Cinnamate target genes were primarily involved in chemical carcinogenesis-receptor activation, lipid and atherosclerosis, the HIF-1 signaling pathway, nitrogen metabolism, and the p53 signaling pathway. Our findings suggested that YBS, palmitic acid, and cinnamate might alleviate LDH levels by regulating metabolism and inflammation, consistent with those of the previous studies.

Palmitic acid has been reported to be beneficial for lumbar fusion between the posterolateral transverse processes by implanting an iliac crest autograft on the fusion bed [39], suggesting that palmitic acid might have a therapeutic effect on LDH. Cinnamate is also known as ferulic acid [40]. Ferulic acid (an active compound from COE) inhibited the DRG inflammatory response in a rat model of nerve injury [41]. However, the potential molecular mechanisms of palmitic acid and cinnamate in LDHs remain unclear. To explore the possible molecular mechanisms of YBS, palmitic acid, and cinnamate in LDH disease, we established a rat LDH model. We obtained nucleus pulposus from an autologous coccyx disc. We applied it to the lumbar nerve root next to the corresponding dorsal root ganglion to establish a rat model of LDH. RNA-Seq was used to analyze DEGs in the DRG tissues of LDH rats. PCA suggested that the control and LDH groups were separated. The results showed that the DEGs were mainly related to cellular processes, biological regulation, regulation of biological processes, metabolic processes, and the PI3K-Akt signaling pathway. It alleviates the symptoms of LDH by increasing cell proliferation and decreasing cell apoptosis and inflammation [42]. LDH might be related to the upregulation of inflammatory mediators [43]. Autophagy is of great significance for the reuptake of LDH [28]. Therefore, we further analyzed significant changes in genes related to metabolism, inflammatory response, autophagy, and apoptosis in LDH. We identified 13 metabolism-related DEGs in LDH. Among them, Gpnmb, Rgs14, and Ncapg expressions were elevated in the LDH model, whereas Stxbp4, CDKN1A, Thbs1, Nupr1l1, Nupr1, Ncapd2, Smoc2, Ccng1, Pkia, and UHRF1 expressions were downregulated in the LDH model. 18 DEGs related to inflammation were identified. Compared with the control group, Ccl19, Lilrb4, Tlr2, S100A9, Adora2a, and Ccl12 expressions increased in the LDH group, whereas SOCS3, B4galt1, Cyp4f6, Cxcl13, Serplnf1, Acp5, Mas1, Ccl9, Wfdc1, Nppb, Trim55, and Apod expressions decreased. Five autophagy-related genes were identified. Epg5 and S100A9 expressions in the LDH group increased, whereas DCN, Dram, and LEPR expressions decreased. Five apoptosis-related DEGs, including Tuba3a and CTSW, were upregulated in the LDH group, and Lmnb1, Ctsk, and BCL2A1 were reduced in the LDH group. The progression of LDH is likely closely related to these differential genes.

Next, we used TNF-*α* to stimulate DRG neuronal cells to establish an *in vitro* LDH model. We used YBS, palmitic acid, and cinnamate for *in vitro* and *in vivo* interventions to explore whether they could alleviate LDH by regulating the expression of metabolism-, inflammation-, autophagy-, and apoptosis-related genes. Studies have shown that *L. paracasei* S16 reduced LDH symptoms by increasing PWT and inhibiting the expression of inflammatory factors (IL-2 and IL-4) [42]. In this study, YBS, palmitic acid, and cinnamate reduced the Siegal neurological score of LDH rats and improved the chances of PWT in the hind limbs after seven days of treatment. After 28 days of treatment, YBS, palmitic acid, and cinnamate reduced the serum IL-1*β* and IL-18 levels. Our findings indicated the effectiveness of YBS, palmitic acid, and cinnamate on the symptoms of LDH in rats. Substance P and CGRP were produced in the dorsal root ganglion neurons and transported to the nerve endings of the spinal dorsal horn, where they played a role in LDH pain transmission [44]. In this study, we found that YBS, palmitic acid, and cinnamate inhibited substance P and CGRP expression. A previous study revealed that cyclinD1, cell division cycle 2 homolog A, and cyclin A2 genes related to the cell cycle were significantly upregulated in the DRG tissues of LDH rats [45]. PCNA and Ki67 positive cells increase in degenerated intervertebral discs [46]. SOCS3 negatively regulates the JAK/STAT3 pathway and alleviates LDH [47]. We found that YBS, palmitic acid, and cinnamate reduced S100A9 and CTSW expressions, while enhancing CDKN1A, UHRF1, PCNA, Ki67, SOCS3, DCN, LEPR, and BCL2A1 expression, as well as telomerase activity in the LDH model DRG tissues. A previous study revealed that cartilage-derived morphogenetic protein-1 (CDMP1) reduced the expression of caspase-3, thereby inhibiting the apoptosis of DRG cells induced by inflammatory cytokines [10]. In the present study, YBS, palmitic acid, and cinnamate reduced the number of cleaved cleaved caspase-3 positive cells in an LDH model. Our results are similar to those of previous studies. Our results suggested that YBS was more effective than palmitic acid or cinnamate. Therefore, we further analyzed the influence of YBS on DEGs in LDH using RNA-Seq technology. PCA suggested that the LDH and YBS groups were well-separated. Due to limited funds, Figure 8 analyzed the RNA-Seq results of the YBS group based on the RNA-Seq results of the control and LDH groups in Figure 2(a). Because of the batch effect of the experiment, the principal component analysis of the control group and the LDH group was similar, and the YBS group was far away from the control group and the LDH group. The results in Figure 8 confirmed the results of WB and further supplemented the analysis of the potential regulatory genes of YBS on LDH, providing a direction for subsequent further research. We found that DEGs mainly involved biological processes, such as cellular processes, biological regulation, metabolic processes, and the pathway of coronavirus disease-COVID-19. YBS downregulated S100A9 and CTSW expressions, while upregulating SOCS3, CDKN1A, UHRF1, DCN, LEPR, and BCL2A1 expressions. The inhibition of the RAGE/STAT3 pathway in the DRG could alleviate persistent pain hypersensitivity caused by LDH [11]. Dexamethasone reduced the LDH levels by restoring the L-PGDS/PI3K/Akt pathway [15]. RvD1 might reduce LDH symptoms by regulating inflammatory mediators and the NF-*κ*B/p65 and p-ERK pathways [48]. The inhibition of the Wnt/*β*-catenin pathway can inhibit DRG neuron apoptosis induced by inflammatory cytokines [10]. Curcumin might inhibit apoptosis of DRG neurons through the AKT/ERK pathway, thereby alleviating the symptoms of LDH [16]. IL-33/ST2 signaling accelerates LDH disease occurrence and progression by regulating MAPK and NF-*κ*B activation [49]. We speculate that YBS, palmitic acid, and cinnamate might alleviate LDH symptoms through these methods. Studies have shown that the YQHR promotes the formation of the Beclin1-VPS34 complex through the upregulation of deubiquitinase USP13 and the activation of AMPK to activate autophagy and inhibit cell apoptosis, thereby alleviating intervertebral disc degeneration development [17]. In this study, we found that autophagy might be negatively related to apoptosis in LDH cells. We suspect that YBS, palmitic acid, and cinnamate might promote the formation of the Beclin1-VPS34 complex through the deubiquitinase USP13/AMPK to activate autophagy and inhibit DRG neuronal cell apoptosis, thereby alleviating LDH symptoms. In subsequent research, we will continue to study the pathway mechanism of YBS, palmitic acid, and cinnamate in LDH treatment and the internal relationship between these pathways. In this project, we obtained the potential regulatory genes of YBS related to inflammation, metabolism, autophagy, and apoptosis in LDH through RNA-Seq sequencing. Considering the limited funds and space, we tested the expression of a few related genes and preliminarily explained the potential regulatory effects of YBS and its key active ingredients palmitic acid and cinnamate on inflammation, metabolism, autophagy, and apoptosis in LDH. Our study has laid a solid foundation for follow-up research. This is the research limitation of this study. In the next project, we will continue to study the regulation and mechanism of YBS and its key active ingredients palmitic acid and cinnamate on other genes related to inflammation, metabolism, autophagy, and apoptosis in LDH, with a view to providing new directions and strategies for clinical treatment of LDH.

YBS might treat LDH by promoting metabolic responses and autophagy and inhibiting inflammation and apoptosis. In addition, Pearson's correlation analysis confirmed a positive correlation between DCN and BCL2A1, suggesting that there might be a negative correlation between autophagy and apoptosis in LDH. YBS, palmitic acid, and cinnamate might inhibit cell apoptosis by activating autophagy in DRG tissues, thereby reducing the symptoms of LDH, which warrants further investigation. Our findings provide a theoretical basis for the reasonable clinical application of YBS, palmitic acid, and cinnamate in treating LDH.

## Figures and Tables

**Table 1:** Chromatographic conditions.

Time (min)	Parameter
0	A: 5%; B: 95%
7	A: 70%; B: 30%
12	A: 100%; B: 0%
13	A: 100%; B: 0%
14	A: 5%; B: 95%
16	A: 5%; B: 95%

A: acetonitrile; B: 0.1% formic acid.

**Table 2:** Primer sequence.

		Primer sequences
*β*-Actin	F: R:	ACATCCGTAAAGACCTCTATGCC TACTCCTGCTTGCTGATCCAC
Substance P	F: R:	ATGAAAATCCTCGTGGCGGT ATCTGACCATGCCCAGCATC
Calcitonin gene-related peptide (CGRP)	F: R:	ATAGCCCCAGAAAGAAGGTTACACA ACAACACGATGCACAATAGCCAAC
CDKN1A	F: R:	TCCTGGTGATGTCCGACCTGTTC GGCTCAACTGCTCACTGTCCAC
UHRF1	F: R:	TCTTCTGCCATTTACGCGGT TGCCGATGTGCTGAAGGAAT
PCNA	F: R:	ATCTAGACGTCGCAACTCCG AGCTGCACTAAGGAGACGTG
Ki67	F: R:	TTCCAGTGAGGAAAGCCACC ACTTAAGGGAGCCACGAAGC
SOCS3	F: R:	CCCGCTTTGACTGTGTACT AAAGGAAGGTTCCGTCGGTG
S100A9	F: R:	AAGCTGCATGAGAACAACCC CAGCCCCAGAACCAAGGTC
DCN	F: R:	CGGTGGCAAATACCCGGATTA AGGGGATTGTCAGGGTCGTA
LEPR	F: R:	TCAAACGTAAGTGGCGCTCT TCTCTGATCCTGCATCCCCA
CTSW	F: R:	GCACCGGAAAGGATCCTCAA TGTGTTTTGATGCGCCACAG
BCL2A1	F: R:	AATCGGCTCCAAGCAAAACG CAGGAGAACACCCCCAAAGG

## Data Availability

The data used to support the findings of this study are available from the corresponding author upon request.

## Supplementary Materials

Supplementary 1 Supplementary Table 1: the MS qualitative analysis results of Yaobishu (YBS).

Supplementary 2 Supplementary Figure 1: HPLC-MS chromatogram of YBS compound. (a) Total ion current in positive ion mode in MS. (b) Total ion current in negative ion mode in MS.

Supplementary 3 Supplementary Figure 2: function analysis of palmitic acid target genes. (a) GO annotation. (b) KEGG pathway analysis.

Supplementary 4 Supplementary Figure 3: cinnamate target gene function analysis. (a) GO annotation. (b) KEGG analysis.

Supplementary 5 Supplementary Figure 4: YBS and its key compound drug concentration screening. (a) CCK-8 was used to evaluate the proliferation ability of normal neuronal cells. (b) CCK-8 was used to evaluate the proliferation of neuronal cells induced by TNF-*α*. (c) Flow cytometry was used to evaluate cleaved caspase-3 expression in neuronal cells induced by TNF-*α*. One-way analysis of variance was used for multiple group statistical analysis. ^∗^*P* < 0.05 vs. control group, ^#^*P* < 0.05 vs. TNF-*α* group.

Supplementary 6 Supplementary Figure 5: CDKN1A, UHRF1, PCNA, and Ki67 protein expressions in DRG tissue were tested by IHC. The magnification was 400×. Scale bar, 25 *μ*m.

Supplementary 7 Supplementary Figure 6: differential gene analysis in RNA-Seq sequencing. (a) Heat map reflected metabolism, inflammation, autophagy, and apoptosis-related gene expression. (b) Statistical analysis of CDKN1A, UHRF1, DCN, LEPR, CTSW, and BCL2A1 mRNA expression.

Supplementary 8 Supplementary Figure 7: the mechanism diagram.

Supplementary 9 Supplementary Figure 8: the original bands of WB from triplicate experiments.
