# Supplementary figures and images for: Yaobishu Regulates Inflammatory, Metabolic, Autophagic, and Apoptosis Pathways to Attenuate Lumbar Disc Herniation
Source: Oxid Med Cell Longev. 2022 May 14;2022:3861380. doi: 10.1155/2022/3861380 (PMC9125431; doi:10.1155/2022/3861380)

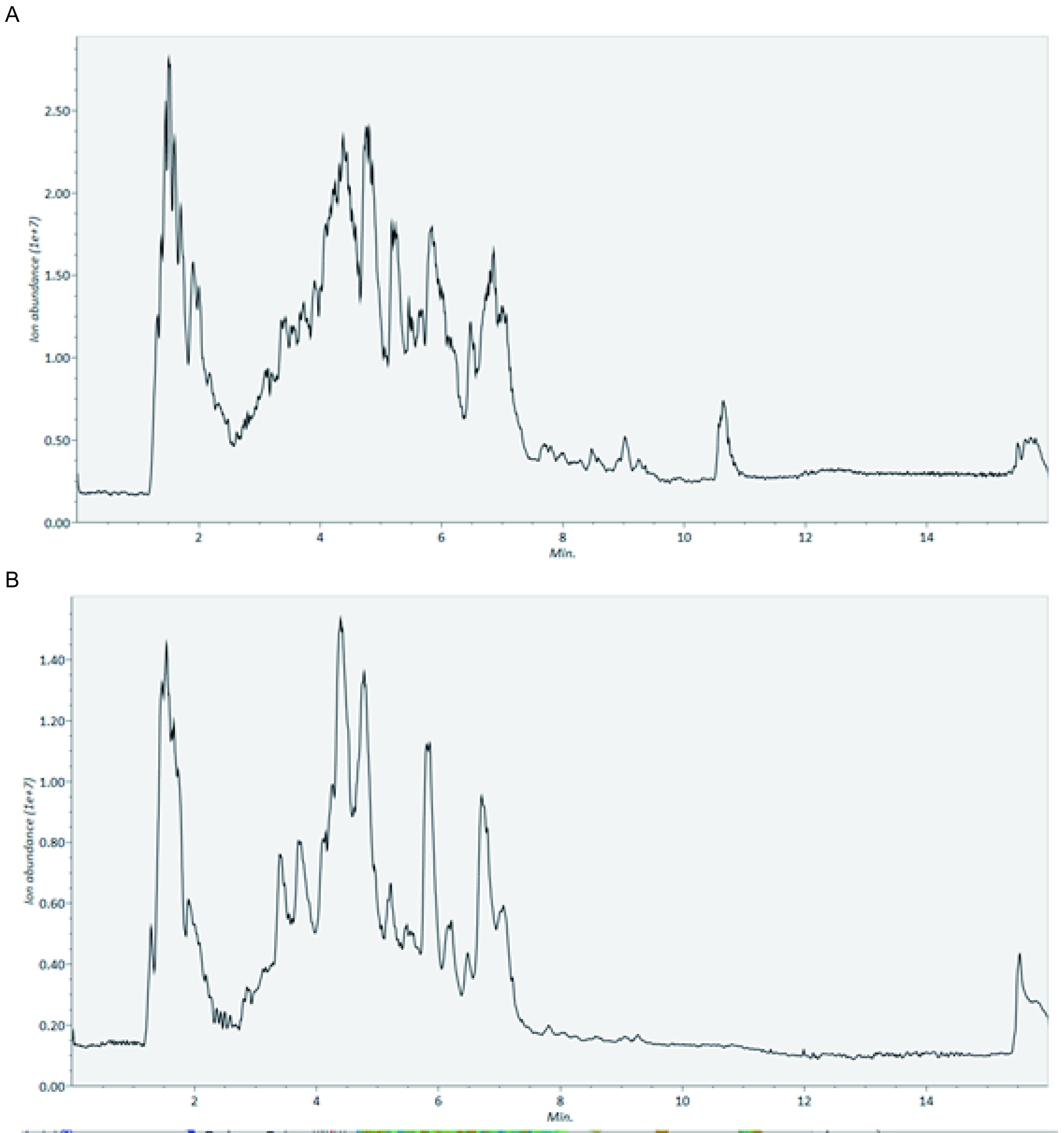

Supplement: Supplementary 2 — Supplementary Figure 1: HPLC-MS chromatogram of YBS compound. (a) Total ion current in positive ion mode in MS. (b) Total ion current in negative ion mode in MS. [file 3861380.f2.jpg]

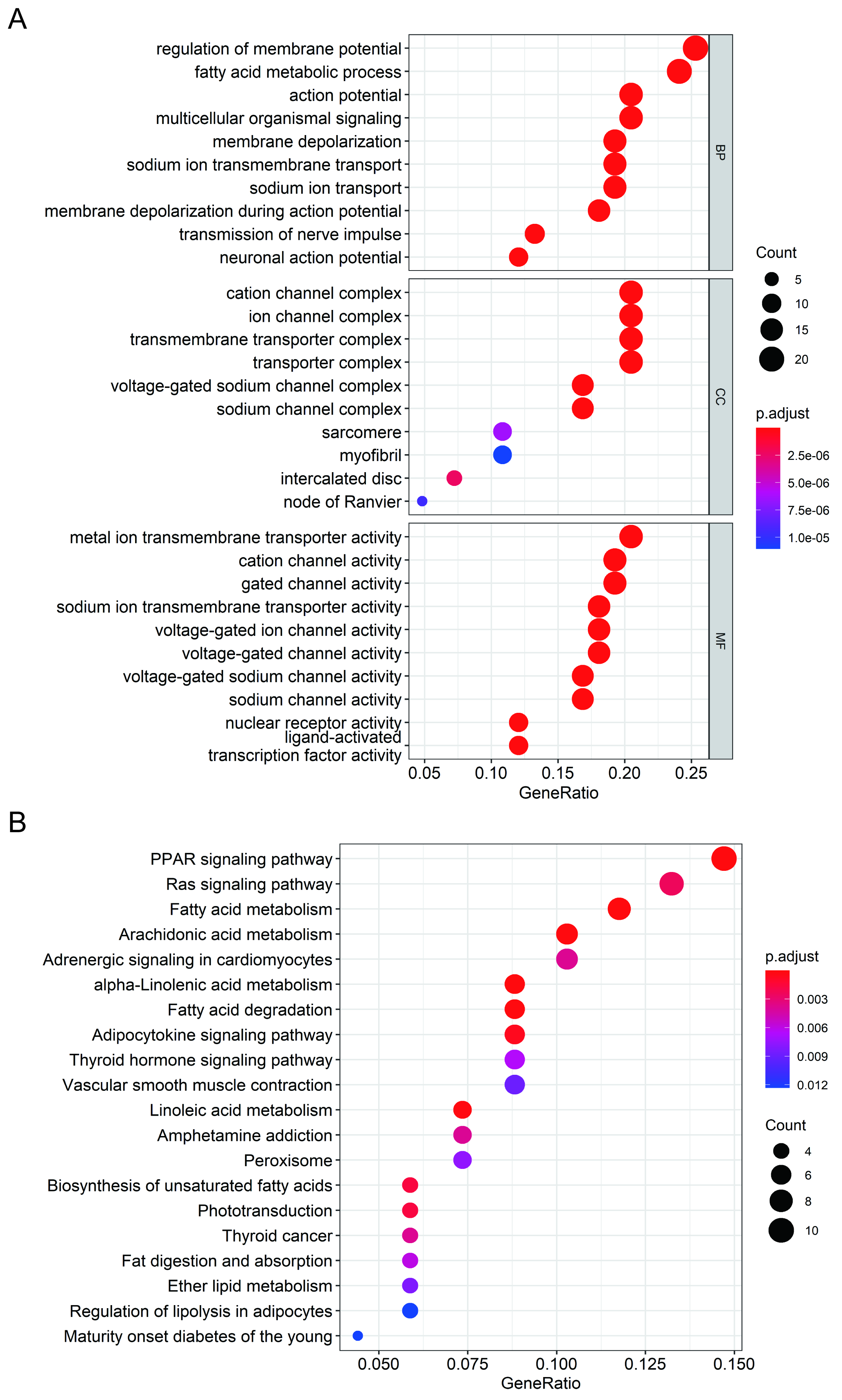

Supplement: Supplementary 3 — Supplementary Figure 2: function analysis of palmitic acid target genes. (a) GO annotation. (b) KEGG pathway analysis. [file 3861380.f3.jpg]

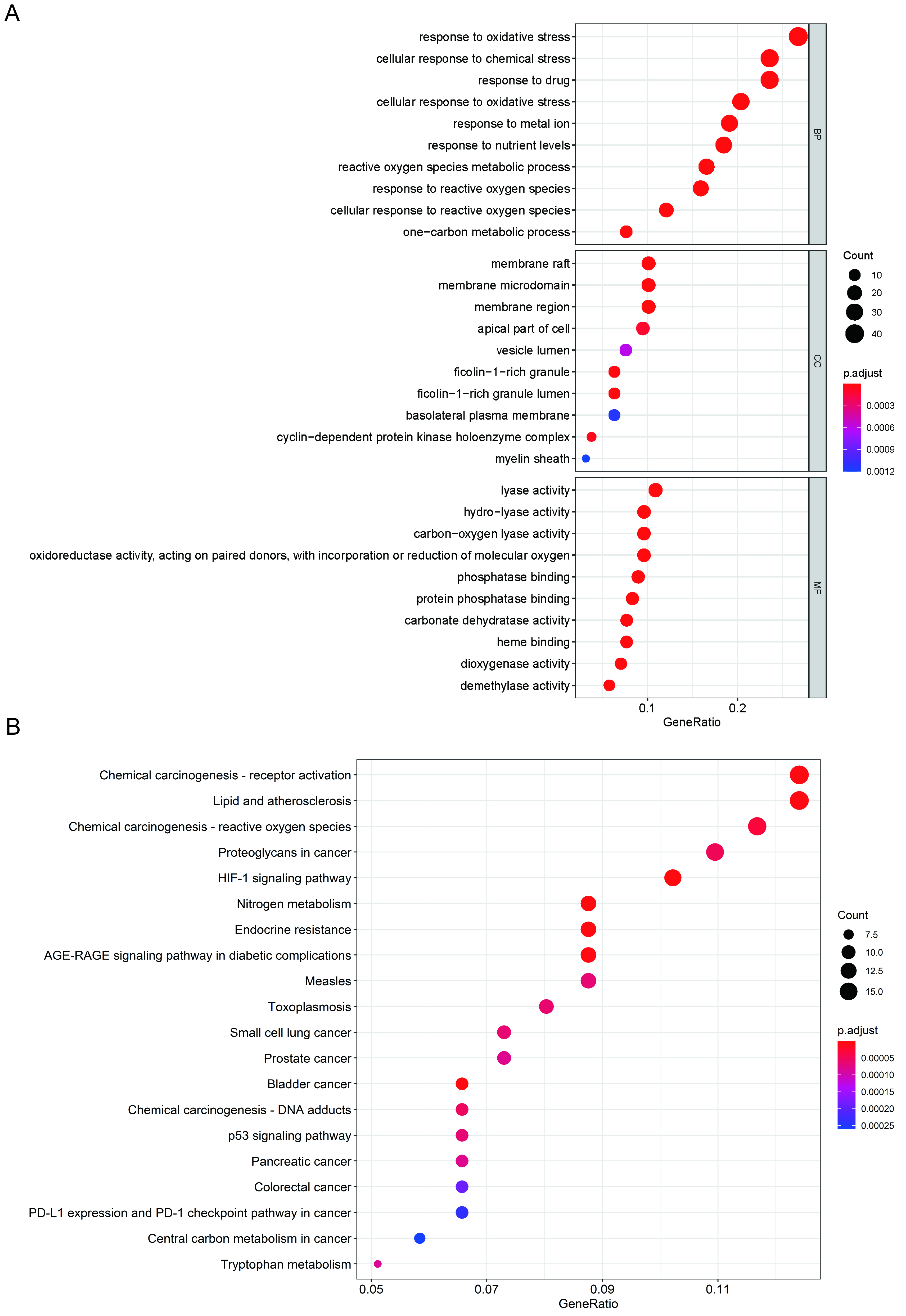

Supplement: Supplementary 4 — Supplementary Figure 3: cinnamate target gene function analysis. (a) GO annotation. (b) KEGG analysis. [file 3861380.f4.jpg]

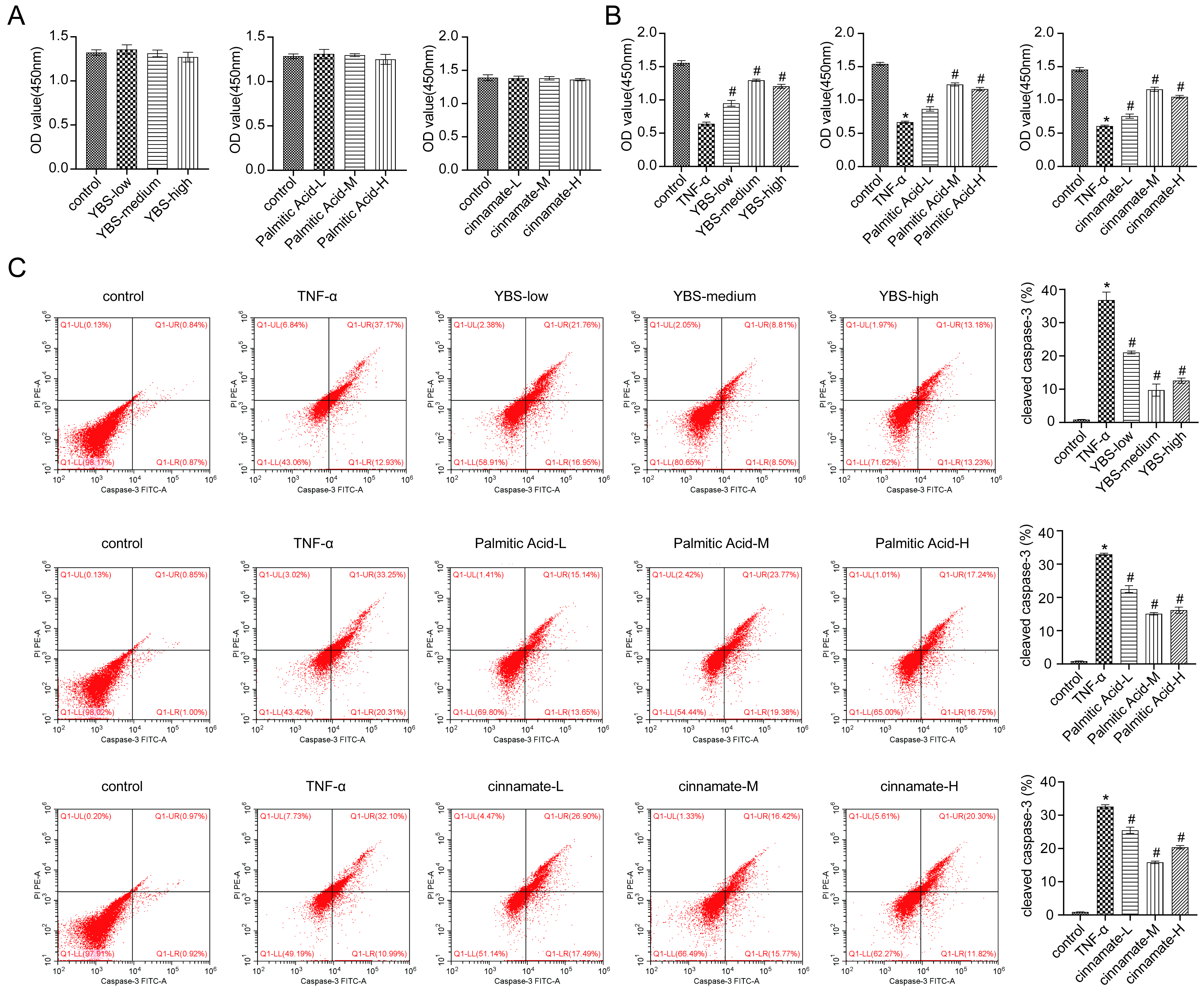

Supplement: Supplementary 5 — Supplementary Figure 4: YBS and its key compound drug concentration screening. (a) CCK-8 was used to evaluate the proliferation ability of normal neuronal cells. (b) CCK-8 was used to evaluate the proliferation of neuronal cells induced by TNF-α. (c) Flow cytometry was used to evaluate cleaved caspase-3 expression in neuronal cells induced by TNF-α. One-way analysis of variance was used for multiple group statistical analysis. ∗P < 0.05 vs. control group, #P < 0.05 vs. TNF-α group. [file 3861380.f5.jpg]

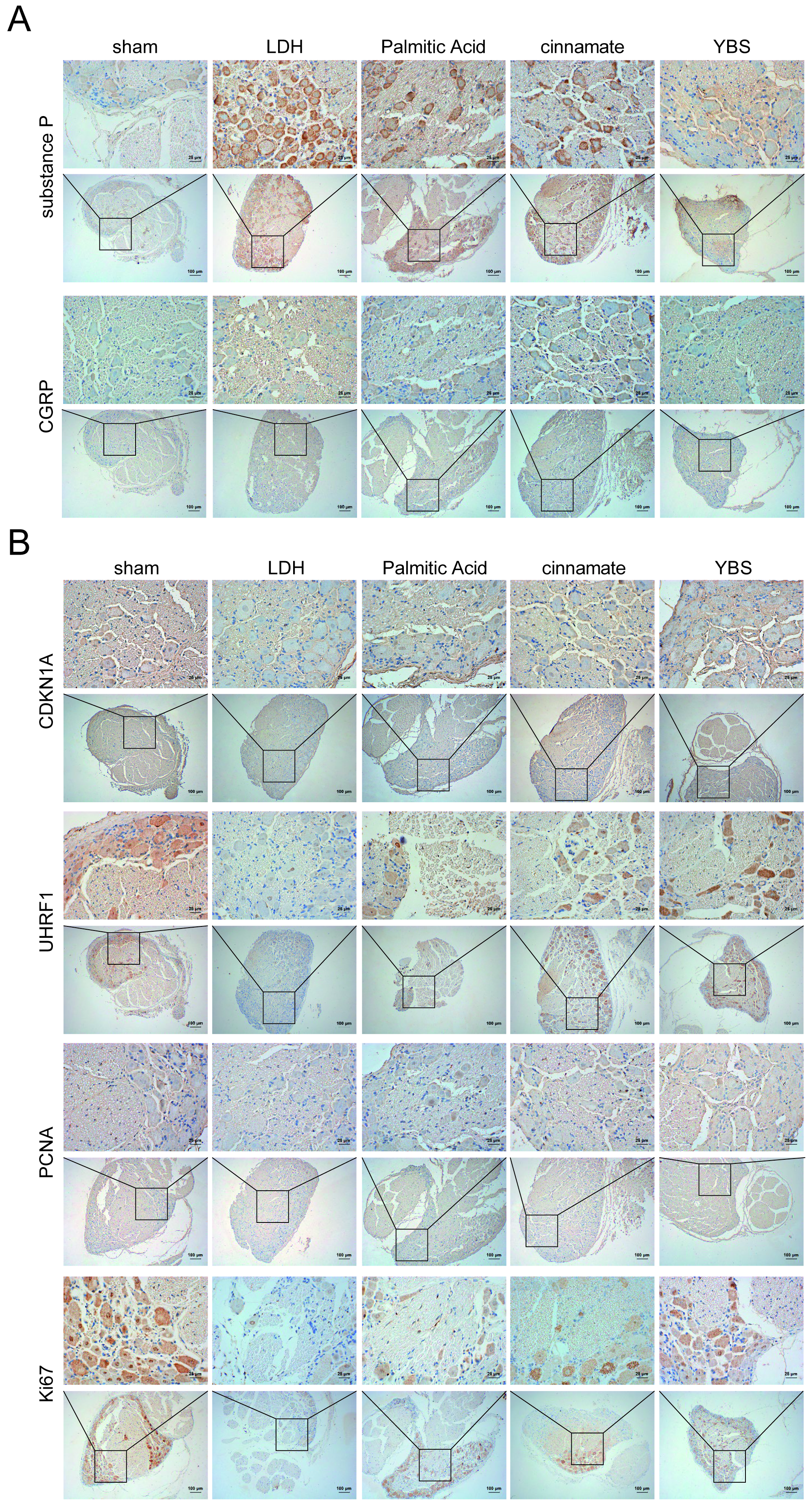

Supplement: Supplementary 6 — Supplementary Figure 5: CDKN1A, UHRF1, PCNA, and Ki67 protein expressions in DRG tissue were tested by IHC. The magnification was 400×. Scale bar, 25 μm. [file 3861380.f6.jpg]

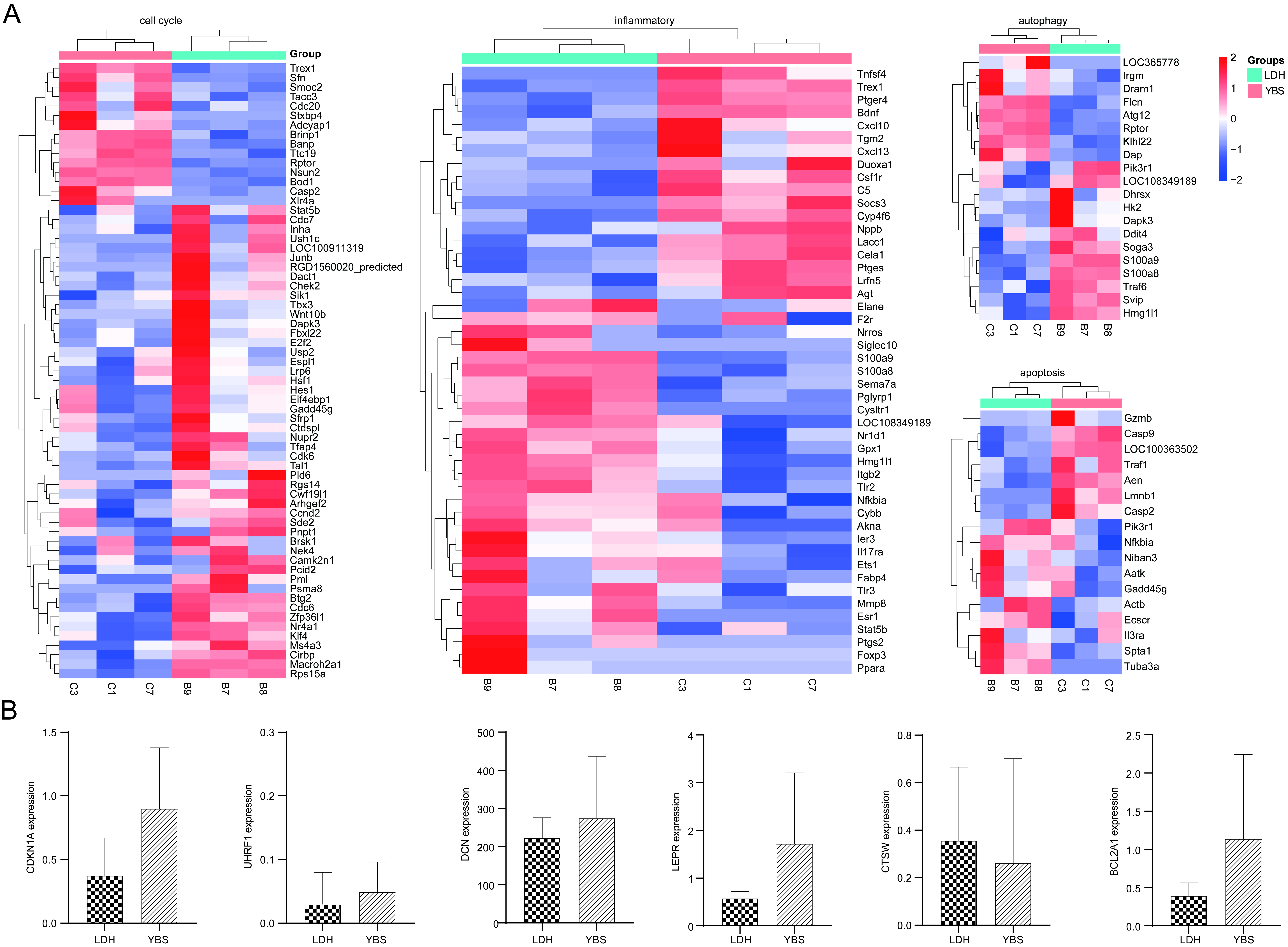

Supplement: Supplementary 7 — Supplementary Figure 6: differential gene analysis in RNA-Seq sequencing. (a) Heat map reflected metabolism, inflammation, autophagy, and apoptosis-related gene expression. (b) Statistical analysis of CDKN1A, UHRF1, DCN, LEPR, CTSW, and BCL2A1 mRNA expression. [file 3861380.f7.jpg]

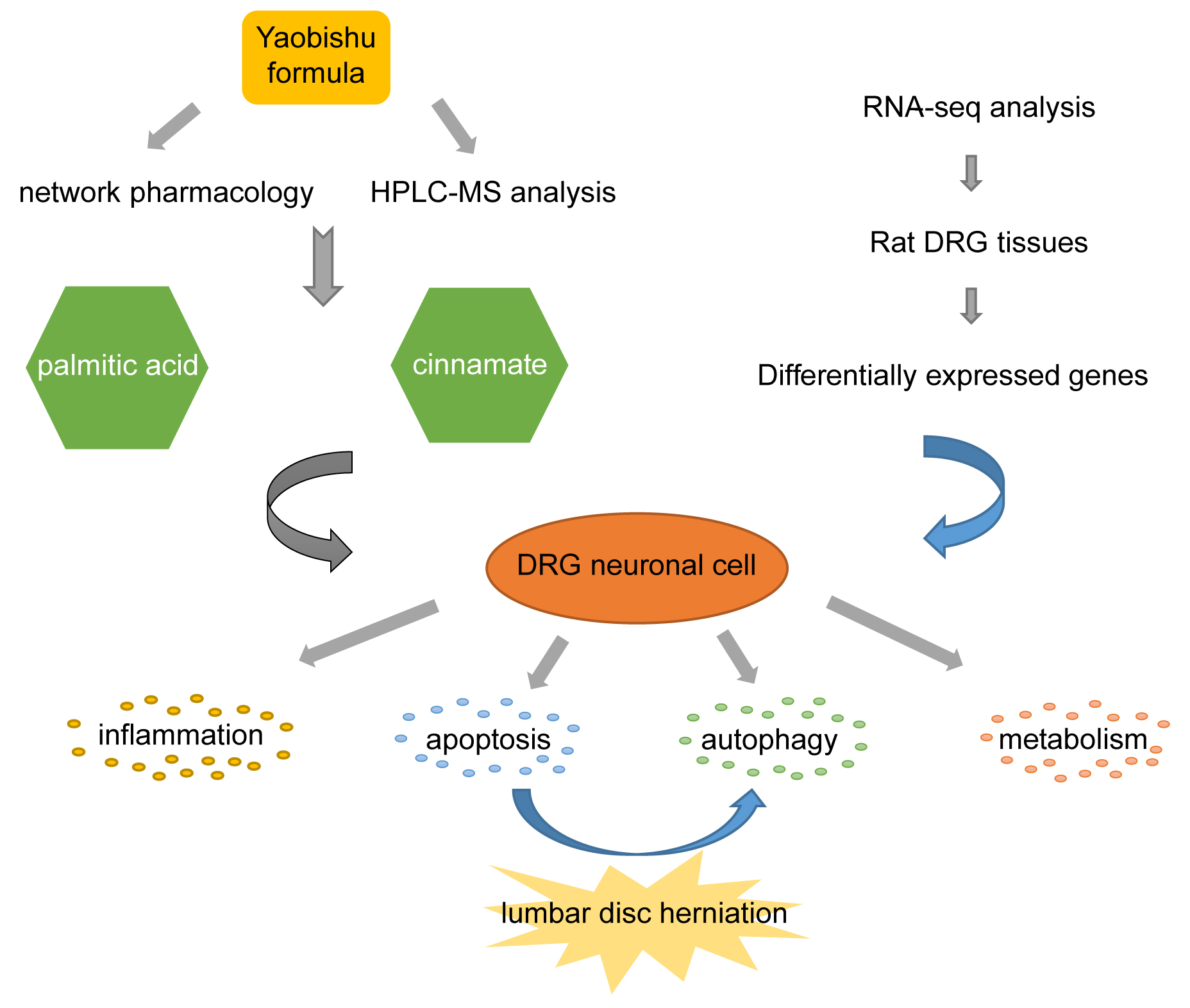

Supplement: Supplementary 8 — Supplementary Figure 7: the mechanism diagram. [file 3861380.f8.jpg]

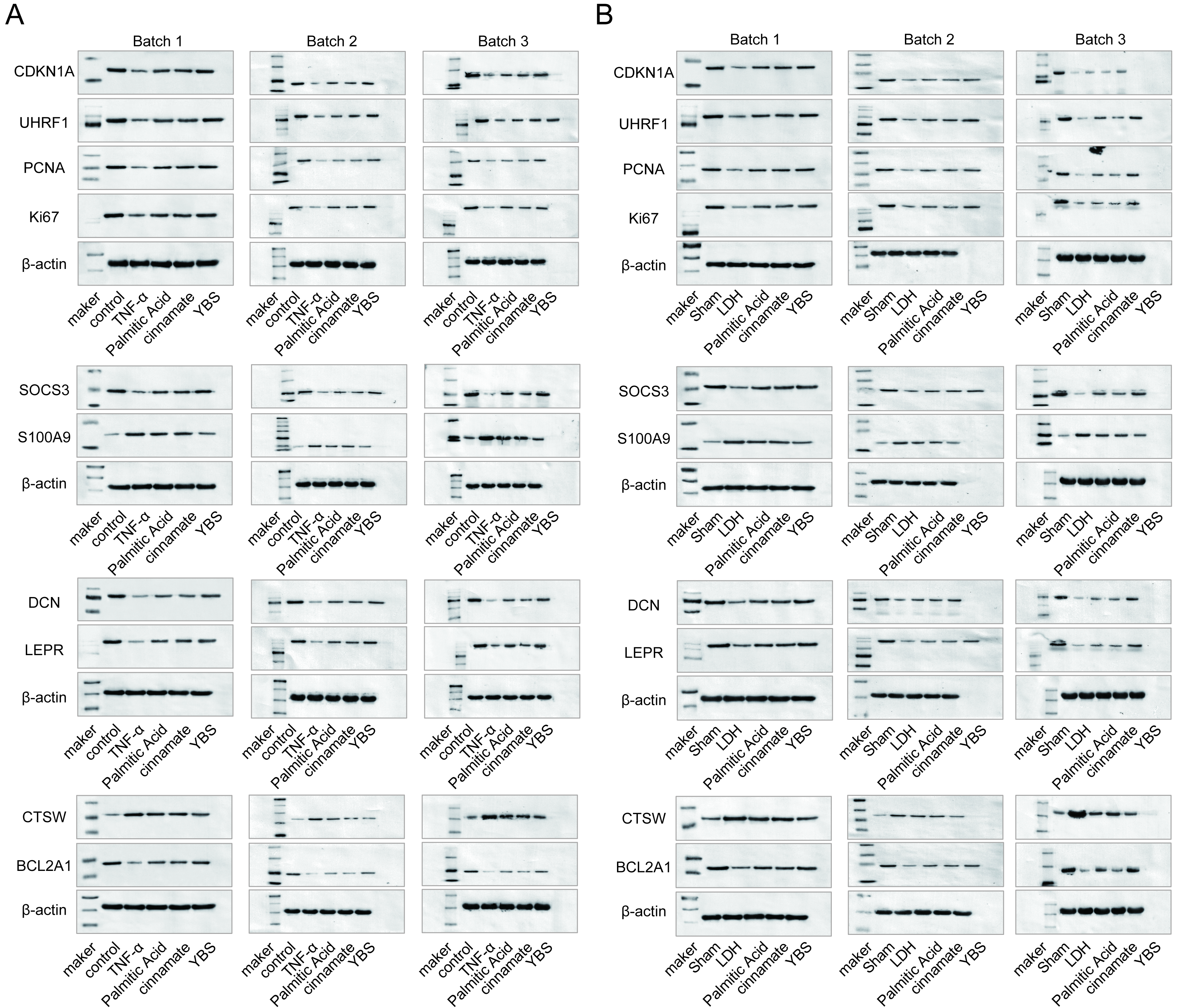

Supplement: Supplementary 9 — Supplementary Figure 8: the original bands of WB from triplicate experiments. [file 3861380.f9.jpg]
